# Identification and Characterization of the Detoxification Genes from the Transcriptome of *Plagiodera versicolora*

**DOI:** 10.3390/insects17060643

**Published:** 2026-06-18

**Authors:** Xiao-Long Liu, Hai-Dong Sun, Yi-Wen Pei, Min Lu, Hai-Nan Zhang

**Affiliations:** 1 State Key Laboratory of Biocatalysis and Enzyme Engineering, School of Life Sciences, Hubei University, Wuhan 430062, China; bruceliu2021@outlook.com (X.-L.L.); shd0909@foxmail.com (H.-D.S.); ppeiyiwen@163.com (Y.-W.P.); lumin@hubu.edu.cn (M.L.); 2 College of Plant Protection, Yangzhou University, Yangzhou 225009, China

**Keywords:** *Plagiodera versicolora*, glutathione S-transferases, UDP-glycosyltransferases, cytochrome P450, carboxylesterases

## Abstract

*Plagiodera versicolora* (Coleoptera: Chrysomelidae), the willow leaf beetle, is a leaf-eating pest that generally occurs on salicaceous trees. However, there is a blank of identification and phylogenetic relationship of the detoxification genes in *P. versicolora*. Here, we identified four detoxification gene families (glutathione S-transferases: GSTs, UDP-glycosyltransferases: UGTs, cytochrome P450 monooxygenases: CYPs and carboxylesterases: COEs) from the adult antennal transcriptome data. In all, 146 candidate detoxification genes including 22 GSTs, 20 UGTs, 60 CYPs, and 44 COEs were identified. We used quantitative real-time PCR technology to explore the tissue expression patterns of 12 PvGSTs in *P. versicolora*. The results showed that 7 PvGSTs have significantly high expression in antennae, indicating these PvGSTs may play an important role in degrade and/or inactivate the sex pheromones and host volatiles. The identification and phylogenetics of the detoxification genes in *P. versicolora* extended the database in Coleoptera and contributed to the subsequent in-depth research for function about detoxification genes.

## Simple Summary

The detoxification system plays an important role in host finding, feeding, and defense against toxic compounds in insects. In this study, four detoxification gene families encoding 146 candidate detoxification genes, including 22 glutathione S-transferases, 20 UDP-glycosyltransferases, 60 cytochrome P450 monooxygenases, and 44 carboxylesterases were identified from the adult antennal transcriptome data in *Plagiodera versicolora*. Then, the expression of the glutathione S-transferases genes was examined using real-time quantitative PCR. These results will provide valuable information to help explore the detoxification mechanism in *P. versicolora*.

## 1. Introduction

In natural environments, insects use complex chemosensory and detoxification systems to find hosts, feed and defend against toxic compounds [1,2,3]. The detoxification system is typically comprised of several genes, mainly including UDP-glycosyltransferases (UGTs), glutathione S-transferases (GSTs), cytochrome P450 monooxygenases (CYPs) and carboxylesterases (COEs) [4,5,6]. These genes also belong to odorant-degrading enzyme family, which is involved in odorant inactivation and xenobiotic processing [7].

GSTs mainly include four families, namely cytosolic GSTs, mitochondrial GSTs and microsomal GSTs [8,9]. Insect cytosolic GSTs are generally classified into six classes, including delta, epsilon, sigma, omega, zeta and theta, with several unclassified classes [10,11]. The amino acid sequences of cytosolic GSTs are usually formed 200 to 250 and are active as either homodimers or heterodimers, whereas microsomal GSTs are typically smaller (about 150 amino acids) and form trimers [12,13,14]. In insects, cytosolic GSTs are generally involved in resistance to insecticides [15,16]. Some GSTs are mainly related to the signal transduction of odor molecules [17].

UGTs in animals are membrane conjugated proteins that occur in the endoplasmic reticulum [18]. UGTs are composed of a variable N-terminus aglycone substrate binding domain, UGT signature motif, and a highly conserved C-terminus UDP-glycoside binding domain. In addition, UGTs generally have a signal peptide (about 20 amino acids) at the N-terminus and a hydrophobic transmembrane domain at the C-terminus, followed by a short cytoplasmic tail [19,20]. Insect UGTs can bind not only a large number of allelochemicals, but also some xenobiotics including insecticides such as chlorpyrifos, imidacloprid [21,22,23].

Cytochrome P450 genes are among the largest families, and are found in almost all organisms, from plants to animals [24,25]. The insect CYPs family can be clustered into four clades: CYP2, CYP3, CYP4, and mitochondrial CYP clades in phylogenetic tree [26]. Insect P450 genes are involved in the acquisition of resistance to a series of toxins, including insecticides and plant-derived toxic compounds [27]. Another important superfamily of COEs also involved in the metabolism of endobiotics and xenobiotics. Insect COEs are divided into three classes of intracellular catalytic, secreted catalytic and neurodevelopmental classes [28,29]. In addition, insect CYPs and COEs play roles in the degradation of pheromone and phytochemical compounds [30,31,32].

The *Plagiodera versicolora* (Coleoptera: Chrysomelidae) mainly feeds on the leaves of salicaceous trees (willows and poplars) [33,34]. To date, no reports are available on the detoxification gene family in *P. versicolora.* In this study, we identified GSTs, UGTs, CYPs, and COEs from the adult antennal transcriptome of *P. versicolora.* We constructed phylogenetic trees and analyzed the phylogenetic relationships of *P. versicolora* with other Coleoptera species. In addition, we analyzed tissue expression levels of GSTs by performing quantitative real-time PCR technology.

## 2. Materials and Methods

### 2.1. Insect Raise and Tissue Collection

In this experiment, the adults of *P. versicolora* were collected from Sha Lake Park in Wuhan, China. In the insect rearing room, relative humidity was maintained at 70 ± 5%, photoperiod was kept at 12 h light: 12 h dark, and the temperature was maintained at 28 ± 1 °C. Different tissues (antennae and bodies) were collected from male and female adults without treatment of chemical or any substances, respectively. All samples were immediately frozen in liquid nitrogen and stored at −80 °C.

### 2.2. Total RNA Extraction and cDNA Synthesis

Total RNA samples from antennae and bodies were isolated using TRIzol Reagent (Thermo Scientific, Waltham, MA, USA, Product code: 15596018CN) following the user guide was supplied by company. The concentration of RNA samples was checked using a NanoDrop-2000 (Thermo Scientific, Waltham, MA, USA). Complementary DNA (cDNA) templates were synthesized using a HiScript III 1st Strand cDNA Synthesis Kit (+gDNA wiper) (Vazyme, Nanjing, China) and stored at −20 °C for gene cloning and expression profiling analysis.

### 2.3. Candidate Gene Identification and Sequence Analysis

Candidate detoxification genes (GST, UGT, P450, and COE) were identified from adult antennal transcriptomes of *P. versicolora* [35]. Subsequently, the candidate detoxification genes were validated using BLASTX (https://blast.ncbi.nlm.nih.gov/Blast.cgi?PROGRAM=blastx&PAGE_TYPE=BlastSearch&LINK_LOC=blasthome (accessed on 6 November 2025)) in the National Center for Biotechnology Information (NCBI) databases. ORF finder (http://www.ncbi.nlm.nih.gov/gorf/gorf.html (accessed on 6 November 2025)) was used to predict the open reading frame (ORFs) of these candidate genes. The SignalP 2.0 server (http://www.cbs.dtu.dk/services/SignalP-2.0/#submission (accessed on 6 November 2025)) was used to predict the signal peptides. The specific names of P450s use the CYP prefix; the family was indicated by an Arabic numeral, and the subfamily was presented with a capital letter. The specific names of GSTs that have a lower-case letter in each subfamily were named according to the classification of insect GSTs. The structure and binding site of GSTs were predicted by searching the InterPro (http://www.ebi.ac.uk/interpro/search/sequence/ (accessed on 6 November 2025)).

### 2.4. Phylogenetic Analysis

Different coleopteran insects were used to construct phylogenetic trees for detoxification gene families. In the P450 tree, four Coleoptera species were selected, including *Xylotrechus quadripes* [36], *Tribolium castaneum* [37], and *P. versicolora.* In the UGT tree, four Coleoptera species were chosen, including *Dendroctonus ponderosae*, *Aethina tumida*, *T. castaneum* [20] and *P. versicolora.* In the GST tree, five Coleoptera were represented, including *X. quadripes*, *Leptinotarsa decemlineata* [38], *Rhaphuma horsfieldi* [36], *T. castaneum* [39], and *P. versicolora.* In the COE tree, four Coleoptera were selected, including *R. horsfieldi*, *X. quadripes*, *Pharsalia antennata* [40], and *P. versicolora.* These protein sequences of detoxification genes were aligned using ClustalX 2.1. Maximum likelihood trees were constructed using FastTree v2.1.11 under the Jones-Taylor-Thornton (JTT) model. The parameter settings are as follows: support values with replicates and CAT approximation with 20 rate categories. In addition, the trees were edited by FigTree v1.4.4 (http://tree.bio.ed.ac.uk/software/figtree/ (accessed on 6 November 2025)). The abbreviation “Pv” is used in the designation of the different genes identified from *P. versicolora* for construction of the phylogenetic tree.

### 2.5. Expression Profiling Analysis

At first, we selected all of 14 full-length PvGSTs to investigate their expression using quantitative real-time PCR (qRT-PCR). The PCR efficiencies of primers ranged from 95–105% with high correlation coefficient (R^2^) values (0.98–0.99) according to the standard. However, two GSTs were substandard primers for qRT-PCR, so we selected 12 PvGSTs for subsequent experiments. We used Beacon Designer 8.14 to design the specific primers (Table S1). The qRT-PCR reaction system was prepared with ChamQ Universal SYBR qPCR Master Mix (Vazyme, Nanjing, China) according to the instructions. The reaction program was set at 95 °C for 30 s, followed by 40 cycles of 95 °C for 5 s and 60 °C for 34 s. Three biological samples for each gene were performed. Gene expression levels were calculated using the 2^−ΔΔCT^ method. The *RPS18* reference gene as the normalization genes was selected to evaluate the relative expression level of PvGSTs. Significant differences were calculated using the ANOVA followed by Tukey’s HSD test with SPSS 26.0 software. Significant differences are indicated by different letters above the bars at a significance level of *p* < 0.05.

## 3. Results

### 3.1. KEGG Pathway Analysis

**Figure 1:**
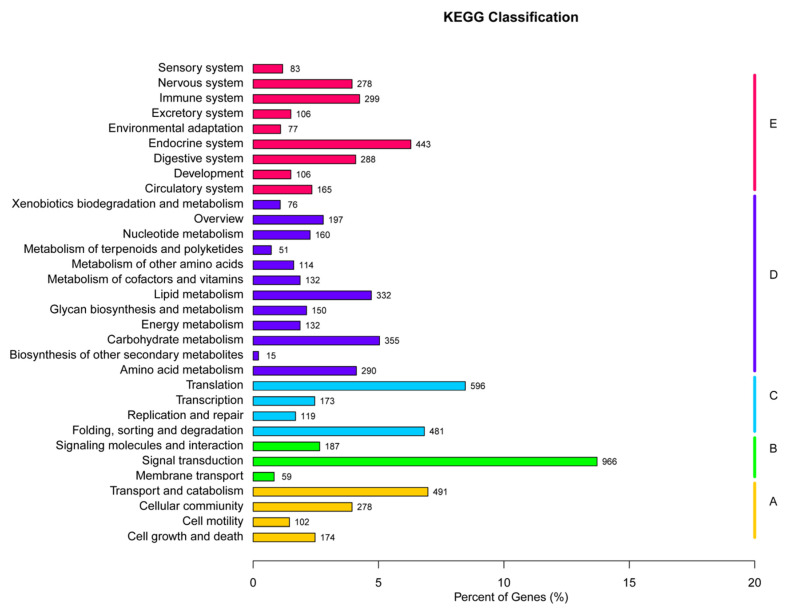
KEGG pathway classification of unigenes in the transcriptome. A: cellular processes; B: environmental information processing; C: genetic information processing; D: metabolism; E: organismal systems.

The unigenes were annotated to 6 databases, including Nucleotide Sequence (NT), Non-Redundant Protein Sequence (NR), Swiss-Prot, Gene Ontology (GO), Kyoto Encyclopedia of Genes and Genomes (KEGG), and clusters of eukaryotic Orthologous Groups (KOG). The numbers of annotated unigenes were 6788 in GO, 3847 in KEGG, 7479 in KOG, 11925 in NR, 3642 in NT, and 8108 in Swiss-Prot databases. The unigenes in the KEGG metabolic pathway were divided into 5 branches: 1045 cellular processes, 1212 environmental information processing, 1369 genetic information processing, 2004 metabolism and 1825 organismal systems (Figure 1).

### 3.2. Identification of GSTs

**Figure 2:**
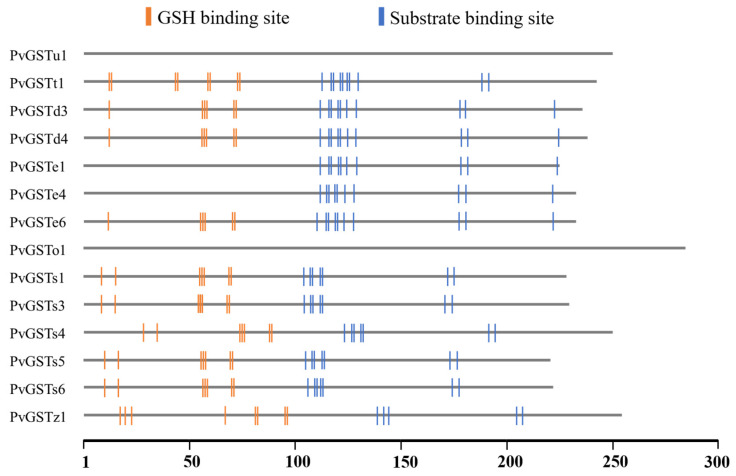
Predicted GSH-binding sites (G-sites) indicated by orange vertical lines and substrate-binding sites (H-sites) indicated by blue lines in PverGST proteins.

**Figure 3:**
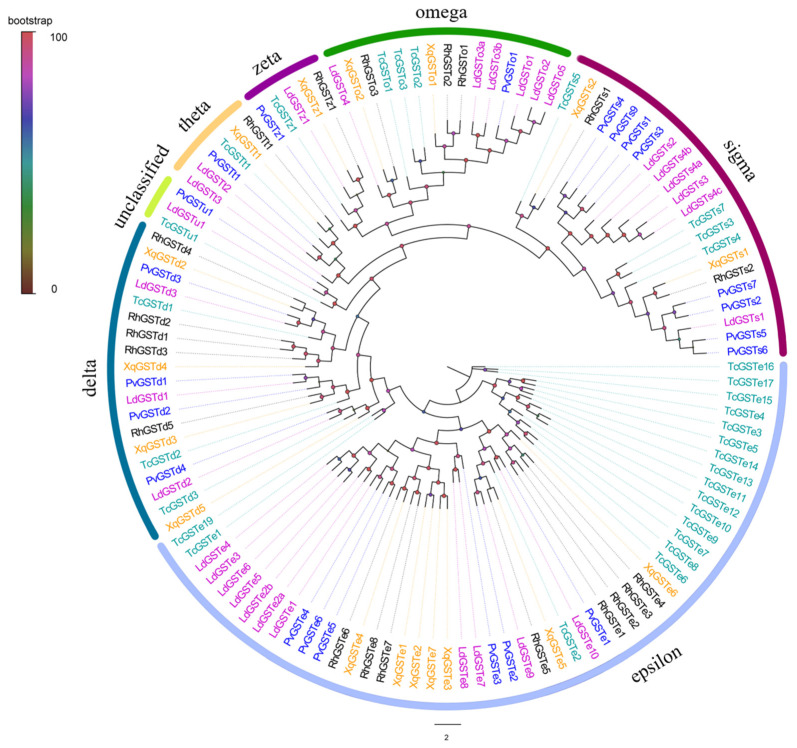
Maximum-likelihood tree of GSTs from *P. versicolora* (Pv), *X. quadripes* (Xq), *L. decemlineata* (Ld)*, R. horsfieldi* (Rh), and *T. castaneum* (Tc). Different species are highlighted with specific color patterns, and *P. versicolora* genes are shown in blue color.

In total, 22 GSTs were identified based on the adult antennal transcriptome of *P. versicolora*. Fourteen PverGSTs had complete ORFs according to the predicted results, and the proteins ranged from 204 to 263 amino acids (Table S2). Conserved domain prediction revealed that 10 PverGSTs had GSH-binding sites and 12 PvGSTs had substrate-binding sites. In addition, two PvGSTs (GSTe1 and GSTe4) had only substrate-binding sites and two PvGSTs (GSTe1 and GSTe4) lacked both sites (Figure 2). All PverGSTs were named according to the phylogenetic results of *P. versicolora* with other Coleoptera species. As a result, the 22 PvGSTs were clustered into eight clades, including epsilon (6 PvGSTs), delta (4 PvGSTs), sigma (8 PvGSTs), zeta (1 PvGST), omega (1 PvGST), theta (1 PvGST) and unclassified classes (1 PvGST) (Figure 3).

### 3.3. Identification of UGTs

**Figure 4:**
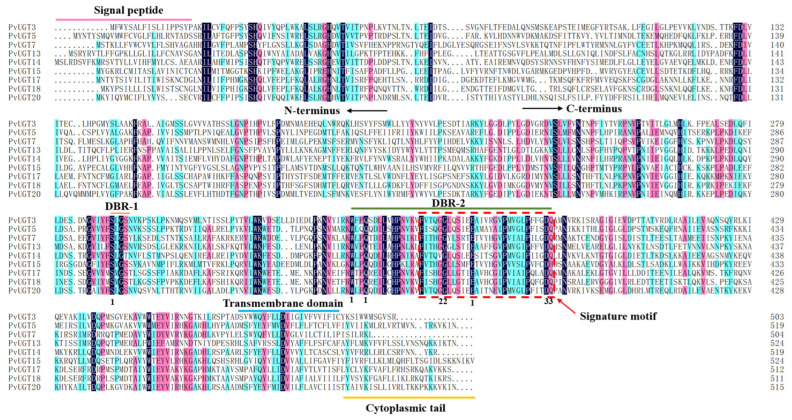
Alignment of the amino acid sequences of 9 full length UGTs. The signal peptides in the N-terminus and the transmembrane domain in C-terminus are marked in pink and blue color at the top, respectively. The signature motif is marked in red box with dashed line. Two donor binding regions (DBR1 and DBR2) are indicated with red and green colored lines at the top of the alignment, respectively. The different shading colors used to highlight homology level of amino acids in the alignment, as pink ≥ 50%, light blue ≥ 75% and dark blue = 100%. The different number of “1”, “2” and “3” are indicated nucleotide-binding residues, phosphate-binding residues and aglycone-binding residues, respectively.

By analyzing the male and female antennal transcriptome dataset, 20 UGTs were identified in *P. versicolora*. Nine PvUGTs had complete ORFs according to the predicted results, and the encoded proteins ranged from 503 to 535 amino acids; the remaining PvUGTs were partial sequences (Table S3). All 9 full-length PvUGT proteins had a signal peptide at the N-terminus and a transmembrane domain at the C-terminus. Of the 9 full-length PvUGT proteins, in the N-terminus, all of PvUGTs had a signal peptide, and these 9 PvUGTs had a transmembrane domain in the C-terminus. Nine PvUGTs have five nucleotide-binding residues, two phosphate-binding residues and two aglycone-binding residues. In addition, sequence analysis showed that two sugar donor-binding regions (DBR1 and DBR2) and a signature motif were also found in these 9 PvUGTs (Figure 4).

**Figure 5:**
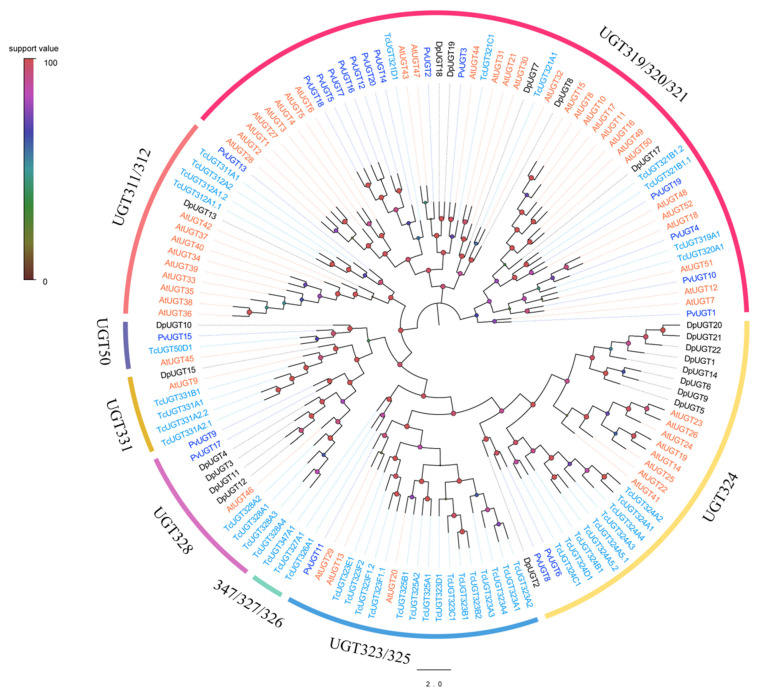
Maximum-likelihood tree of UGTs from *P. versicolora* (Pv), *D. ponderosae* (Dp), *A. tumida* (At), and *T. castaneum* (Tc). The different species are highlighted with specific color patterns, and *P. versicolora* genes are shown in blue color.

Phylogenetic tree results showed that the 20 UGTs were clustered into eight subfamilies: UGT50, UGT311/312, UGT319/320/321, UGT324, UGT323/325, UGT347/327/326, UGT328 and UGT331. No PverUGTs were found in the UGT331 or UGT347/327/326 subfamilies. Only one member of PvUGT11, PvUGT13 and PvUGT15 were found in the UGT323/325, UGT311/312 and UGT50 subfamilies, respectively. PvUGT9/UGT17 and PvUGT6/UGT8 were clustered into the UGT328 and UGT324 subfamilies, respectively. The UGT319/320/321 subfamilies formed the largest cluster and included PvUGT1, UGT2, UGT3, UGT4, UGT5, UGT7, UGT10, UGT12, UGT14, UGT16, UGT18, UGT19 and UGT20 (Figure 5).

### 3.4. Identification of CYPs

**Figure 6:**
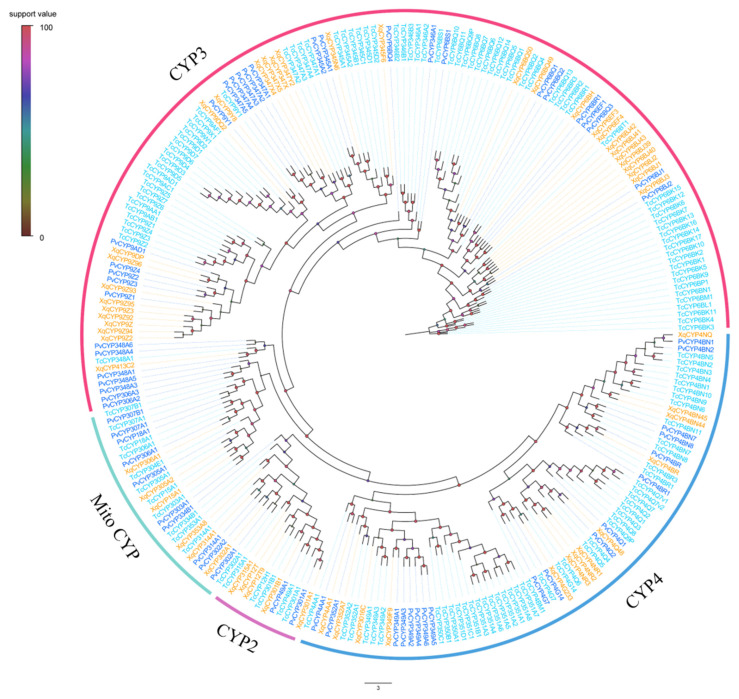
Maximum-likelihood tree of CYPs from *P. versicolora* (Pv), *T. castaneum* (Tc), and *X. quadripes* (Xq). Different species are highlighted with specific color patterns, and *P. versicolora* genes are shown in blue color.

We identified a total of 60 CYPs from the adult antennal transcriptome of *P. versicolora*. Among the CYP genes, 28 PvCYPs had complete ORFs according to the predicted result, and the protein ranged from 463 to 579 amino acids (Table S4). The remaining 32 PvCYPs were partial sequences due to the absence of 5- or 3-terminals. The results of phylogenetic analysis suggested that CYP genes were clustered into four families: mitochondrial (Mito) members (10 PvCYPs), CYP2 (2 PvCYPs), CYP3 (30 PvCYPs) and CYP4 (18 PvCYPs) (Figure 6).

### 3.5. Identification of COEs

**Figure 7:**
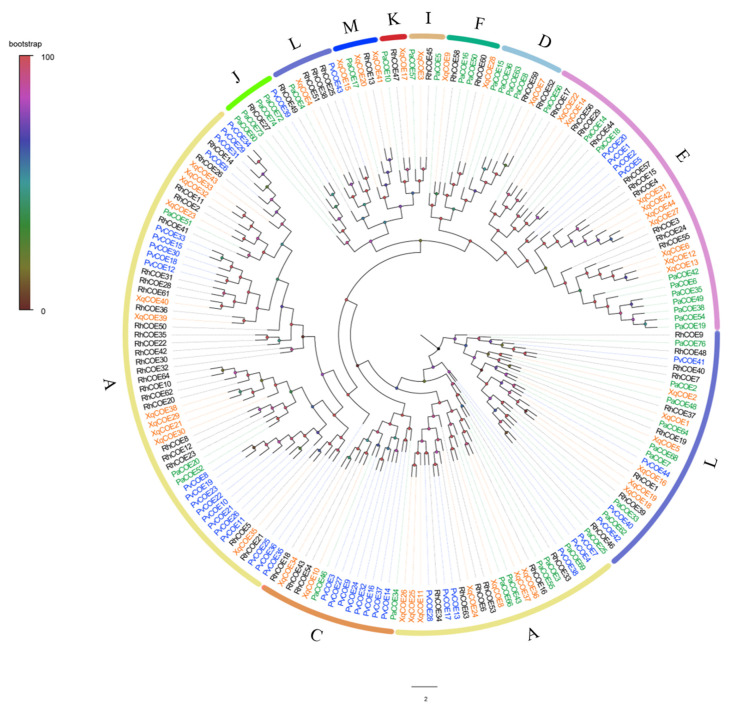
Maximum-likelihood tree of COEs from *P. versicolora* (Pv), *R. horsfieldi* (Rh), *P. antennata* (Pa), and *X. quadripes* (Xq). Different species are highlighted with specific color patterns, and *P. versicolora* genes are shown in blue. Intracellular catalytic class: A: Coleopteran xenobiotic metabolizing enzymes; C: Microsomal and α-esterases. Secreted catalytic class: D: Integument esterases; E: β- and pheromone esterases; F: Non-lepidopteran juvenile hormone esterases; Neurodevelopmental class: I: Uncharacterized group; J: Acetylcholinesterases; K: Gliotactins; L: Neuroligins; M: Neurotactins.

In all 44 COEs were obtained from the transcriptome and 19 PvCOEs have complete ORFs following the predicted result (Table S4). According to the classification system of insect COEs, PvCOEs were grouped into three large classes and 5 clades. Phylogenetic tree results showed that clade A had the largest number of COEs, with 26 PvCOEs, and clade C had 8 PvCOEs in the intracellular catalytic class. The clade E had 4 PvCOEs in the secreted catalytic class, whereas clade L had 5 PvCOEs and clade J had 1 PvCOE in neurodevelopmental class. In addition, no PvCOEs were grouped into the remaining five clades (D, F, I, K, M) (Figure 7).

### 3.6. Expression Profile of GSTs

**Figure 8:**
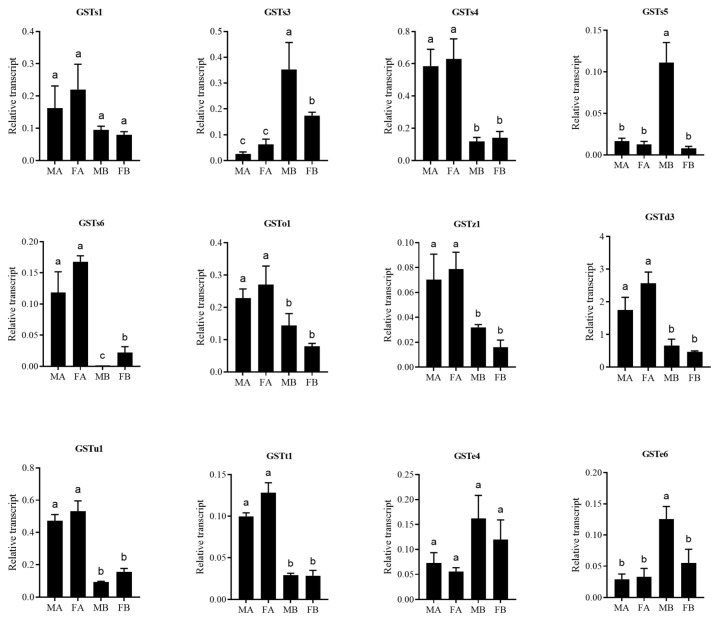
Expression levels of PvGST genes in different tissues determined by qRT-PCR. MA, male antennae; FA, female antennae; MB, male body without antennae; FB, female body without antennae. Error bars indicate the standard error of three biological replicates. The different letters (a–c) indicate significant differences in different tissues.

To explore the expression profile of GSTs, antennae and bodies were selected and tested by qRT–PCR (Figure 8). PvGSTs2 and PvGSTe4 showed no significant difference between antennae and bodies of male and female. PvGSTs3, PvGSTs5 and PvGSTe6 were highly expressed in bodies than in antennae, and further analysis showed that these 3 PvGST were highly expressed in male bodies than in female bodies. PvGSTs4, PvGSTs6, PvGSTo1, PvGSTz1, PvGSTd3, PvGSTu1 and PvGSTt1 had higher expression levels in antennae than in bodies. In addition, expression level of these 7 PvGSTs showed no bias in male and female antennae.

## 4. Discussion

The adult antennal transcriptome of *P. versicolora* was analyzed, and several detoxification genes were identified in this study. The number of PvGST genes (22 members) was comparable to the number found in the genome and transcriptome of *Bombyx mori* (23 members) [41], less than 30 GSTs in *L. decemlineata* [38] and 41 GSTs were identified in *T. castaneum* [39] based on the whole genome database and NCBI, more than 14 GSTs in *Rhodnius prolixus* [42] and 10 GSTs in *Apis mellifera* [5]. The number of PvUGTs (20 members) observed is similar to those found in *Holotrichia parallela* (20 members) [43], less than 30 UGTs in *X. quadripes* [44] and more than 13 UGTs in *Ostrinia furnacalis* [45].

We identified 60 PvCYPs from the antennal transcriptome. This number was lower than that reported in *P. antennata* (64 CYP genes) [40], *A. tumida* (116 CYP genes) [46], *D. ponderosae* (85 CYP genes) [47]. This possibly indicates that more CYP genes may be obtained through further identification in *P. versicolora*. In addition, 44 PvCOEs were identified from the antennal transcriptome. The number is same as *X. quadripes* (44 COEs), less than 64 COEs in *R. horsfieldi* and 72 COEs in *L. decemlineata*, and more than 35 COEs in *Drosophila melanogaster* [5]. Meanwhile, these results suggested that the number of detoxification genes varies greatly among different insect species. Pesticides are chemicals that control different pests. These detoxification enzymes (GSTs, UGTs, CYPs and COEs) can reduce the toxicity of pesticides contributed to insect physiology and developmental progress [48].

In the phylogenetic tree of UGTs, some species-specific expansions appeared in *P. versicolora*, PvUGT319/320/321 subfamily possessed 13 members, including PvUGT1, UGT2, UGT3, UGT4, UGT5, UGT7, UGT10, UGT12, UGT14, UGT16, UGT18, UGT19, and UGT20. Similar patterns were found in other Coleopteran insects, including 8 UGTs of UGT352 in *X. quadripes* [44], 15 UGTs of UGT352 in *P. antennata* [49], 8 UGTs of UGT324 in *T. castaneum* [20], 9 UGTs of UGT312 in *A. tumida*, and 9 UGTs of UGT324 in *D. ponderosae*. The results of phylogenetic analyses indicated that this UGT subfamily may have undergone a tandem duplication event.

Unlike odorant-binding proteins and odorant receptor play the fundamental role in the detection and transduction of diverse odor molecules [50]. GSTs that have a highly expressed level in antennae are more likely to degrade and/or inactivate sex pheromones and host volatiles. For example, SzGSTd1 has a specific expression level in the antennae of *Sitophilus zeamais* and SzGSTd1 functions to selectively degrade capryl alcohol (stored grain volatile) [51]. In the Lepidoptera, many studies have focused on the function of antennae-specific GSTs. PiGSTd1 was antennae-specific and further analysis revealed that PiGSTd1 could degrade and inactivate Z9-12:Ac (sex pheromone component) and aldehyde (host volatiles) of *Plodia interpunctella* [52]. BmGSTD4 had a specific expression in antennae and a high GSH-binding capacity to 1-chloro-2, 4-dinitrobenzene in *B. mori* [53]. An antenna specific GST-msolf1 could degrade trans-2-hexenal (green leaf volatile) in *Manduca sexta* [54]. The qRT-PCR results showed that the expression levels of 7 PvGSTs (GSTs4, GSTs6, GSTo1, GSTz1, GSTd3, GSTu1 and GSTt1) were higher in antennae than in bodies. Thus, it is suggesting that 7 PvGST may be associated as odorant degradation enzyme to ensure the sensitivity of the olfactory system.

## 5. Conclusions

In conclusion, we obtained 146 detoxification genes from the adult antennae of *P. versicolora* transcriptome data, including 22 GSTs, 20 UGTs, 60 CYPs, and 44 COEs. Expression profiles showed that 3 PvGST were highly expressed in abdomens, indicating that these PvGSTs may be involved in substance metabolism. In addition, 7 PvGST have high expression levels in the antennae, suggesting that these PvGSTs may take part in odorant perception. This research will be helpful for exploring the detoxification and chemoreception mechanisms of *P. versicolora* in the future.

## Data Availability

The data presented in this study are available in this manuscript; further inquiries can be directed to the corresponding author. The raw reads of the transcriptomes in this study have been submitted in the NCBI SRA database, with the BioProject of PRJNA1471247.

## Supplementary Materials

The following supporting information can be downloaded at: https://www.mdpi.com/article/10.3390/insects17060643/s1, Table S1: Primers for qRT-PCR of GSTs in *P. versicolora*. Table S2: The Blastx match of *P. versicolora* GST genes. Table S3: The Blastx match of *P. versicolora* UGT genes. Table S4: The Blastx match of *P. versicolora* CYP genes. Table S5: The Blastx match of *P. versicolora* COE genes. File S1: The amino acid of GST, UGT, CYP and CXE in the male and female antennae transcriptome of *P. versicolora*.
