# Supplementary material for: Identification and Characterization of the Detoxification Genes from the Transcriptome of Plagiodera versicolora
Source: Insects. 2026 Jun 18;17(6):643. doi: 10.3390/insects17060643 (PMC13301284; doi:10.3390/insects17060643)
Supplement: Supplementary file 1 [file insects-17-00643-s001.zip › insects-4381496-supplementary.pdf]

Supplementary data

Table S1. Primers for qRT-PCR of GSTs in *P. versicolora*.

GSTs1-F	ACCTTCACTACCTCTAGGACAA	GSTs1-R	CCAGCGTTGCCACATATCTA
GSTs3-F	GCAGAGCCAATGAGGATGAT	GSTs3-R	GTAGTTGCCCAAAGGGTAGAG
GSTs4-F	ATGTGCCTGGAAGCGATAAA	GSTs4-R	CCCTCCACGATTTCCCATAAA
GSTs5-F	GCGCATCAAAGTATCGCTATC	GSTs5-R	GTATCGACGGCAGAGTCTATTT
GSTs6-F	TCGGCAACTACAGATACGAAAG	GSTs6-R	ATCCAGTTTCCCGAGGTAGTA
GSTo1-F	CGGAACGATCGGGATGTATTT	GSTo1-R	CGGCTGTTGACTCATGTCTT
GSTz1-F	GAAGGAGGGTTGGGTGAAAT	GSTz1-R	CACGCAATCCGCTAAAGTTATG
GSTd3-F	TCAATCCTCAACACACCATACC	GSTd3-R	CTTGGGATACAGGGTGTCATTT
GSTu1-F	ATGTGCCTGGAAGCGATAAA	GSTu1-R	CCCTCCACGATTTCCCATAAA
GSTt1-F	ATCACGGTTTGGTGGGTATG	GSTt1-R	CACAGCCTCCTCATAGAGAAAC
GSTe4-F	CGAATGCATCCGTTTACGTTTC	GSTe4-R	CTTCGTTCAGGTCACTGTATGG
GSTe6-F	GGACTGAAACTAGACGTGGTATATT	GSTe6-R	AGGGTTGGTACTGTGTGTTTAG
q18S-F	CTTCCTCGTCGGAGCATTCT	q18S-R	GTTCGCCTTAACTGCCATCAA

Table S2. The Blastx match of *P. versicolora* GST genes.

Name	ORF (aa)	Complete	Blast best hit ACC. NO. Gene Species	E-value	Identity (%)
GSTd2	148	N	QYA72011.1|glutathione S-transferase [Anoplophora glabripennis]	4e-52	56.08
GSTd1	62	N	APX61025.1|putative glutathione S-transferase delta class member 1 [Leptinotarsa decemlineata]	8e-33	68.42
GSTd3	218	Y	APX61027.1|putative glutathione S-transferase delta class member 3 [Leptinotarsa decemlineata]	3e-130	82.33
GSTe1	208	Y	QWJ89647.1|glutathione S-transferase e2 [Sitophilus zeamais]	2e-79	59.02
GSTe2	159	N	AVT42186.1|glutathione S-transferase e2 [Lissorhoptrus oryzophilus]	2e-47	65.91
GSTe3	114	N	UNY86279.1|glutathione S-transferase epsilon1 [Phyllotreta striolata]	3e-55	73.68
GSTe4	215	Y	APX61028.1| putative glutathione S-transferase epsilon class member 1 [Leptinotarsa decemlineata]	1e-84	57.41
GSTe5	147	N	APX61028.1|putative glutathione S-transferase epsilon class member 1 [Leptinotarsa decemlineata]	2e-39	45.07
GSTe6	215	Y	APX61028.1|putative glutathione S-transferase epsilon class member 1 [Leptinotarsa decemlineata]	4e-71	50.00
GSTo1	263	Y	APX61040.1|putative glutathione S-transferase omega class member 2 [Leptinotarsa decemlineata]	8e-125	67.35
GSTs1	211	Y	APX61046.1|putative glutathione S-transferase sigma class member 2 [Leptinotarsa decemlineata]	1e-80	56.93
GSTs2	144	N	QWV59559.1|glutathione S-transferase sigma 2 [Lasioderma serricorne]	2e-40	68.00
GSTs3	213	Y	APX61046.1|putative glutathione S-transferase sigma class member 2 [Leptinotarsa decemlineata]	2e-71	60.71
GSTs4	231	Y	QWV59564.1|glutathione S-transferase [Lasioderma serricorne]	2e-133	78.26
GSTs5	204	Y	APX61045.1|putative glutathione S-transferase sigma class member 1 [Leptinotarsa decemlineata]	1e-101	65.84
GSTs6	205	Y	AIL23548.1|glutathione S-transferase sigma [Tenebrio molitor]	2e-95	66.50
GSTs7	109	N	APX61045.1|putative glutathione S-transferase sigma class member 1 [Leptinotarsa decemlineata]	1e-43	64.22
GSTt1	225	Y	APX61052.1|putative glutathione S-transferase theta class member 2 [Leptinotarsa decemlineata]	7e-103	62.84
GSTu1	232	Y	KAG5864165.1|Glutathione S-transferase [Gonioctena quinquepunctata]	3e-82	55.50
GSTd4	220	Y	WET52671.1|glutathione S-transferase [Phaedon brassicae]	2e-140	90.91
GSTz1	235	Y	APX61057.1|putative glutathione S-transferase zeta class member 1 [Leptinotarsa decemlineata]	4e-97	62.93
GSTs9	130	N	KAG5864165.1|Glutathione S-transferase [Gonioctena quinquepunctata]	3e-44	48.17

Table S3. The Blastx match of *P. versicolora* UGT genes.

Name	ORF (aa)	Complete	Blast best hit<br>ACC. NO. Gene Species	E-value	Identity (%)
UGT1	102	N	KAI7815625.1|UDP-glucuronosyltransferase [Rhyzopertha dominica]	2e-23	52.5
UGT2	121	N	AVT42221.1|UDP-glucuronosyltransferase 321E1 [Lissorhoptrus oryzophilus]	2e-31	60.4
UGT3	503	Y	KAI7815289.1|UDP-glucuronosyltransferase [Rhyzopertha dominica]	8e-164	50.32
UGT4	110	N	QIK00360.1|UDP-glycosyltransferase [Xylotrechus quadripes]	2e-29	47.62
UGT5	519	Y	RZC39915.1|UDP-glucuronosyltransferase 2B20-like [Asbolus verrucosus]	1e-158	48.94
UGT6	85	N	XP_023020371.1|UDP-glucuronosyltransferase 2B15-like [Leptinotarsa decemlineata]	8e-24	81.54
UGT7	504	Y	QIK00369.1|UDP-glycosyltransferase [Xylotrechus quadripes]	0.0	54.76
UGT8	124	N	QIK00374.1|UDP-glycosyltransferase [Xylotrechus quadripes]	3e-10	48.10
UGT9	69	N	XP_045473179.1|UDP-glucosyltransferase 2-like isoform X2 [Harmonia axyridis]	2e-08	55.10
UGT10	150	N	KAI7815625.1|UDP-glucuronosyltransferase [Rhyzopertha dominica]	2e-51	55.56
UGT11	271	N	QIK00372.1|UDP-glycosyltransferase [Xylotrechus quadripes]	5e-102	61.14
UGT12	138	N	KAI7815289.1|UDP-glucuronosyltransferase [Rhyzopertha dominica]	2e-44	55.48
UGT13	524	Y	QIK00370.1|UDP-glycosyltransferase [Xylotrechus quadripes]	8e-157	47.63
UGT14	519	Y	RZC39915.1|UDP-glucuronosyltransferase2B20-like [Asbolus verrucosus]	7e-166	50.94
UGT15	525	Y	QIK00361.1|UDP-glycosyltransferase [Xylotrechus quadripes]	0.0	78.37
UGT16	89	N	QIK00372.1|UDP-glycosyltransferase [Xylotrechus quadripes]	3e-15	56.76
UGT17	512	Y	QIK00367.1|UDP-glycosyltransferase [Xylotrechus quadripes]	2e-172	50.38
UGT18	511	Y	QIK00367.1|UDP-glycosyltransferase [Xylotrechus quadripes]	8e-165	51.31
UGT19	108	N	RZC35969.1|UDP-glucuronosyltransferase 2B7 [Asbolus verrucosus]	2e-25	47.62
UGT20	515	Y	UNY86281.1|UDP-glucuronosyltransferase 2 [Phyllotreta striolata]	0.0	56.31

Table S4. The Blastx match of *P. versicolora* CYP genes.

Name	ORF (aa)	Com plete	Blast best hit ACC. NO. Gene Species	E-value	Identity (%)
CYP18A1	529	Y	UNY86268.1|CYP18a1 [Phyllotreta striolata]	0.0	78.83
CYP306A2	202	N	UNY86270.1|CYP306a1 [Phyllotreta striolata]	7e-44	55.48
CYP306A3	147	N	AGT57833.1|cytochrome P450 306a1 [Leptinotarsa decemlineata]	8e-34	69.05
CYP301A1	521	Y	QZM07456.1|cytochrome P450 CYP301A1 [Lasioderma serricorne]	0.0	78.03
CYP302A1	100	N	KYB24706.1|Cytochrome P450 6a2-like Protein [Tribolium castaneum]	1e-10	37.70
CYP302A2	511	Y	AGT57842.1|cytochrome P450 302a1 [Leptinotarsa decemlineata]	0.0	66.60
CYP303A1	493	Y	RZC40009.1|cytochrome P450 303a1 [Asbolus verrucosus]	0.0	59.22
CYP305A1	463	Y	AGT57832.1|cytochrome P450 305a1 [Leptinotarsa decemlineata]	3e-180	56.73
CYP307A1	97	N	KAI7815183.1|cytochrome p450 [Rhyzopertha dominica]	1e-27	89.66
CYP307B1	217	N	EFA11558.2|cytochrome P450 307A1 [Tribolium castaneum]	3e-127	75.85
CYP314A1	489	Y	UYL69089.1|cytochrome P450 314a1 [Colaphellus bowringi]	0.0	70.35
CYP345A1	500	Y	QYA71972.1|cytochrome P450 [Anoplophora glabripennis]	0.0	53.51
CYP345A2	502	Y	QYA71972.1|cytochrome P450 [Anoplophora glabripennis]	0.0	56.46
CYP346A1	92	N	AZR39430.1|cytochrome P450 [Agasicles hygrophila]	4e-29	48.28
CYP347A1	140	N	AGT57862.1|cytochrome P450 347c1 [Leptinotarsa decemlineata]	4e-46	54.68
CYP347A2	278	N	AGT57862.1|cytochrome P450 347c1 [Leptinotarsa decemlineata]	1e-118	62.93
CYP347A3	366	N	QHO62281.1|CYP347W1 [Phaedon cochleariae]	0.0	60.00
CYP347A4	497	Y	QHO62281.1|CYP347W1 [Phaedon cochleariae]	0.0	88.31
CYP347A5	494	Y	QHO62281.1|CYP347W1 [Phaedon cochleariae]	2e-160	49.59
CYP348A1	340	N	AGT57838.1|cytochrome P450 413a1 [Leptinotarsa decemlineata]	8e-85	48.97
CYP334B1	65	N	ABG20821.1|cytochrome P450 [Leptinotarsa decemlineata]	7e-46	70.00
CYP348A3	223	N	UYL69089.1|cytochrome P450 314a1 [Colaphellus bowringi]	5e-88	74.86
CYP348A4	81	N	AZR39479.1|cytochrome P450 [Agasicles hygrophila]	4e-32	57.89
CYP348A5	235	N	QLL22071.1|cytochrome P450 [Tribolium confusum]	1e-134	62.57
CYP348A6	111	N	KRT85019.1|cytochrome P450 [Oryctes borbonicus]	9e-45	47.10
CYP349A1	245	N	QYA71990.1|cytochrome P450 [Anoplophora glabripennis]	1e-97	64.47
CYP349A2	363	N	AZR39479.1|cytochrome P450 [Agasicles hygrophila]	7e-160	59.01
CYP349A3	500	Y	XP_023023382.1|cytochrome P450 4C1-like [Leptinotarsa decemlineata]	0.0	57.50
CYP349A4	496	Y	AFI45011.1|cytochrome P450 CYP349b1 [Dendroctonus ponderosae]	2e-109	43.19
CYP349A5	178	N	QYA71990.1|cytochrome P450 [Anoplophora glabripennis]	1e-19	36.61
CYP349A6	506	Y	QYA71967.1|cytochrome P450 [Anoplophora glabripennis]	3e-135	43.12
CYP352A1	522	Y	AZR39478.1|cytochrome P450 [Agasicles hygrophila]	4e-172	53.35
CYP306A1	303	N	AGT57833.1|cytochrome P450 306a1 [Leptinotarsa decemlineata]	3e-108	63.55
CYP49A1	354	N	UUB32621.1|cytochrome P450 CYP49A1 [Dendroctonus rhizophagus]	0.0	81.79
CYP4AA1	485	Y	AZR39454.1|cytochrome P450 [Agasicles hygrophila]	0.0	54.62
CYP4BN1	71	N	UZE89825.1|cytochrome P450 CYP4XE1 [Chrysoperla zastrowi sillemi]	2e-29	57.32
CYP4BN2	101	N	ABF06551.1|CYP4BH1 [Ips paraconfusus]	1e-24	56.18
CYP4BN7	416	N	QYA71991.1|cytochrome P450 [Anoplophora glabripennis]	7e-153	52.06’
CYP4BN8	486	Y	QYA71991.1|cytochrome P450 [Anoplophora glabripennis]	7e-152	46.79

CYP4BR	423	N	AZR39469.1|cytochrome P450 [Agasicles hygrophila]	0.0	69.89
CYP4BR1	439	N	KAG5891040.1|Cytochrome P4504C1 [Gonioctena quinquepunctata]	2e-107	67.77
CYP4G14	549	Y	AAZ94273.1|cytochrome P450 [Leptinotarsa decemlineata]	0.0	87.34
CYP4G7	579	Y	ANS06314.1|cytochrome P450 [Agasicles hygrophila]	0.0	68.57
CYP4Q1	504	Y	AZR39463.1|cytochrome P450 [Agasicles hygrophila]	0.0	61.17
CYP4Q2	152	N	AKZ17712.1|cytochrome P450 monooxygenase CYP4Q33 [Tenebrio molitor]	7e-33	60.34
CYP6BJ1	101	N	AAZ94272.1|cytochrome P450 [Leptinotarsa decemlineata]	6e-67	74.24
CYP6BJ2	427	N	AJA91072.1|cytochrome P450 [Leptinotarsa decemlineata]	0.0	66.45
CYP6BQ1	514	Y	AGT57849.1|cytochrome P450 6bq15 [Leptinotarsa decemlineata]	0.0	67.14
CYP6BQ2	364	N	AZR39427.1|cytochrome P450 [Agasicles hygrophila]	0.0	54.56
CYP6BQ3	89	N	AIE17439.1|cytochrome P450 CYP6P9a [Anopheles funestus]	8e-10	61.22
CYP6BQ4	516	Y	AZR39435.1|cytochrome P450 [Agasicles hygrophila]	0.0	60.94
CYP6BR1	518	Y	QEG78945.1|CYP6BH5 [Phaedon cochleariae]	0.0	80.45
CYP6BS1	520	Y	AZR39433.1|cytochrome P450 [Agasicles hygrophila]	0.0	76.08
CYP6EF1	564	Y	AGT57858.1|cytochrome P450 6ef1 [Leptinotarsa decemlineata]	0.0	56.58
CYP9AD1	522	Y	AAZ94269.1|cytochrome P450 [Leptinotarsa decemlineata]	0.0	58.20
CYP9Y1	479	Y	RZC41760.1|cytochrome P450 9e2-like [Asbolus verrucosus]	0.0	56.08
CYP9Z1	428	N	AJA91073.1|cytochrome P450 [Leptinotarsa decemlineata]	0.0	66.36
CYP9Z2	253	N	KAI4464070.1|cytochrome p450 [Holotrichia oblita]	4e-59	38.16
CYP9Z3	533	Y	AZR39445.1|cytochrome P450 [Agasicles hygrophila]	6e-170	55.13
CYP9Z4	512	Y	AZR39449.1|cytochrome P450 [Agasicles hygrophila]	0.0	54.05

Table S5. The Blastx match of *P. versicolora* COE genes.

Name	ORF (aa)	Complete	Blast best hit ACC. NO. Gene Species	E-value	Identity (%)
COE1	600	Y	RZC33123.1|venom carboxylesterase-6-like [Asbolus verrucosus]	5e-140	45.11
COE2	551	Y	KAI4470761.1|carboxylesterase [Holotrichia oblita]	4e-130	46.61
COE3	199	N	UNY86275.1|carboxylesterase type B [Phyllotreta striolata]	3e-76	56.98
COE4	316	N	AKZ17679.1|carboxylesterase COE19 [Tenebrio molitor]	8e-40	79.37
COE5	570	Y	KAI4470761.1|carboxylesterase [Holotrichia oblita]	3e-135	46.26
COE6	366	N	XP_023018363.1|venom carboxylesterase-6-like [Leptinotarsa decemlineata]	1e-155	60.05
COE7	557	Y	KAG6795358.1|palmitoleoyl-protein carboxylesterase NOTUM [Apis mellifera caucasica]	0.0	70.64
COE8	449	Y	WEQ60889.1|esterase [Phaedon brassicae]	4e-154	53.44
COE9	532	Y	AIY68380.1|putative alpha-esterase [Leptinotarsa decemlineata]	0.0	52.15
COE10	321	N	WEQ60889.1|esterase [Phaedon brassicae]	3e-95	48.77
COE11	565	Y	XP_023027565.1|venom carboxylesterase-6-like	0.0	53.45
COE12	612	Y	WEM02070.1| esterase [Phaedon brassicae]	0.0	57.35
COE13	416	N	WEQ60881.1| esterase [Phaedon brassicae]	0.0	75.91
COE14	517	Y	XP_023025748.1|esterase B1-like [Leptinotarsa decemlineata]	0.0	72.46
COE15	310	N	WEP24378.1|venom carboxylesterase [Phaedon brassicae]	9e-88	78.75
COE16	439	N	XP_050515675.1|juvenile hormone esterase-like	5e-126	57.02
COE18	598	Y	WEM02069.1| esterase [Phaedon brassicae]	0.0	59.64
COE19	493	Y	WEQ60889.1| esterase [Phaedon brassicae]	1e-173	51.50
COE20	257	N	WEM02079.1| carboxylesterase [Phaedon brassicae]	2e-159	83.27
COE21	120	N	WET52656.1|cholinesterase [Phaedon brassicae]	1e-33	58.88
COE22	508	Y	WEP24376.1|esterase [Phaedon brassicae]	1e-102	74.27
COE23	386	N	WET52656.1|cholinesterase [Phaedon brassicae]	8e-105	45.80
COE24	353	N	WEQ60888.1|esterase [Phaedon brassicae]	5e-172	66.10
COE25	363	N	WEQ60886.1|esterase [Phaedon brassicae]	2e-135	83.87
COE26	365	N	WEQ60889.1|esterase [Phaedon brassicae]	3e-111	46.13
COE27	556	Y	AIY68380.1|putative alpha-esterase	0.0	51.04
COE28	476	Y	WEM02082.1| esterase [Phaedon brassicae]	0.0	69.53
COE29	383	N	XP_023018362.1|venom carboxylesterase-6-like [Leptinotarsa decemlineata]	0.0	65.21
COE30	198	N	WEM02085.1| esterase [Phaedon brassicae]	8e-92	75.41
COE31	177	N	XP_023018364.1|venom carboxylesterase-6-like [Leptinotarsa decemlineata]	4e-60	60.84
COE32	158	N	XP_028131086.1|esterase B1 [Diabrotica virgifera virgifera]	1e-52	59.0
COE33	437	N	ASM90853.1|putative juvenile hormone esterase 2 [Colaphellus bowringi]	0.0	60.87
COE34	158	N	WEP24377.1|esterase [Phaedon brassicae]	6e-82	79.25
COE35	228	N	XP_050504131.1|juvenile hormone esterase isoform X6 [Diabrotica virgifera virgifera]	3e-73	53.68
COE36	510	Y	XP_023020292.1|esterase E4-like [Leptinotarsa decemlineata]	0.0	54.97
COE37	372	N	XP_023025748.1|esterase B1-like [Leptinotarsa decemlineata]	2e-140	58.17
COE38	737	Y	XP_023024615.1|neurotactin [Leptinotarsa decemlineata]	0.0	68.65
COE39	627	Y	AEI70752.1|acetylcholinesterase 2 [Leptinotarsa decemlineata]	0.0	90.17

COE40	336	N	XP_023020261.1|neuroligin-4, X-linked-like [Leptinotarsa decemlineata]	0.0	81.69
COE41	156	N	XP_060517417.1|neuroligin-4, Y-linked [Cylas formicarius]	3e-59	88.79
COE42	117	N	XP_023011840.1|neuroligin-1-like [Leptinotarsa decemlineata]	3e-41	72.73
COE43	777	Y	XP_015835928.1|neuroligin-1 [Tribolium castaneum]	0.0	72.47
COE44	1041	Y	XP_056632908.1|neuroligin-4, X-linked-like [Diorhabda ublineata]	0.0	57.36

File S1. The amino acid of GST, UGT, CYP and CXE in the male and female antennae transcriptome of *P. versicolora*.

GST

>PvGSTu1

MKLTLYAVSDGPPSLAVRQALAYLKLPHELINVDFGSGDHMTEEFEKKNPQKEIPVLDDDGFFL
GESNAILQYLADKYGKDSTLYPSDPKSRAIVNHRLCFNLSTYYRYISEYSMAPIFFDYQRTPLAL
RKVKIALDNYNTYLKRLGCRYAAGNSITIADFQLVTATMCLEAINFNINEWPLVDLWYKTFKSE
YPNLWEIVEGGMKEISEFEKNPPDLSHMDHPIHPVKRR

>PvGSTu2

MLETEAIKEQSAIVKLFPHTKSFPSRKTSRNPNASSKGPLSSSLIISSPKNSTGKQMALILGYNVP
QSKYSLWLTSALLLGSLGYKLSSFPYVVAR

>PvGSTt1

MVLKLYYDLLSQPCRALYIFLKITKTPFEACVVNLMKGEHLSKKFEQECSRFKKVPFIHHNSFR
LTESVSILRYIAREFPIEDHWYPKDSQKQAEVDEYLEWHHSNVRQPCLTYFRNQWIIPLRTGKE
PSQEDSTKDKNNMLKSLKDFENLFLTDMDFIHGNEISFSDLQAACEIEQSRFGGYDPRKDFPKI
KAWIERVRVECNPYYDEAHSFLYEEAVKKISQ

>PvGSTd2

MKYLSTQYGKDDSLYPTNPKMAALVDLRLFFCATYLFPRLVAFHVPTMFYGSPPNEENRNKLE
ESLQALDKFLENQKWVAGEHMNKNGLEQTITIIASIEAAETFDISTFSNIWSWYQSSKSAMESF
GYEEVCQKGAEAFGTAYKSQLK

>PvGSTd1

MVNHTLIFYHFHMSPVSRAALLLTRALGIKHTVETVNILAKEQMNPNFIKVSDRTSQRIFRL

>PvGSTd3

MPIDFYYVPGSAPCRNVLLAAKAVGVELNLKYTDLMKGEHLTPEFLKINPQHTIPTIDDNGFAL
WESRAIMTYLQEQYGKNDTLYPKDPKQRALVDQRLYFDMGTLYGSFAEYYYPVIFGNASYDP
AKLEKINGAFKFLDQFIGDNDYCTGKNLTLADLSLVSTVSTFEVMDFDLSPYKNVTRWLAKVK
ATAPGYEEANGKPVLAFKQLVETLLKNKK

>PvGSTe1

MLVLHGTSTSPAVRSCLLTLHALGVEFTQNPVNILAGEHLLPQYLKINPLHTIPTLQDGETTIFDS
HAINTYLVDRYGKGDDSLYPNDLTQRAVVNQRLFFDCGTLFTAFRNVVVQILKMGAKVVAKD
HALAINEAYDSLETLMEGSTYVVGQNLTVADLSIISTVTSMNVFVPIASNRYPSITKWMGLMQE
LPYYQKANQEGLEVFT

>PvGSTe2

MGLTLYYAEVSSAARGSLLLIKALGLEVELKPVNLMTGEHMTPEYLAMNPFHTVPTLRDGDF
VIWDSQAINIYLAEKYGKDSPLYPNDPQKRAVIHQRLFFHSGILFPRVSAIVGSLLREGAKSVAK
DKADSVTEGRYILGVFDMSLKFKTNLRGIENA

>PvGSTe3

MVVSLYMIPASPPVRAVRMCAKAIGVDLNLINVDLFSGDHLKPEYLKINPQHTVPLLDDDGFIV
ADSHAIMSYLVSKYGKDKSLYPTDLQQRAIVDHRLHFDSSILFTRGVVIS

>PvGSTe4

MAPKLYAVDLSPCVRAVILCAKALSLELDVIETNLLKKDHLKPEFLRMNPQHTVPTLDDDGVIV
WDSHAIMIYLVDKYGNGNSIYPKNLVERAKINQRLFFDTDLFSKSCRITELVIFEGLKVVPENLK
VPLIKCFEILEKFLESSVYIAGNQLTIADFSLWTTITNASVYVSVDPGKYPRLAAWIKKLESLPYS

DLNEVGGRKFAELISSLLTK

>PvGSTe5

MIYLAEKYGKGNPIYPQDVKRRALVNLRLFFDGQDLFGQHKSVIFPLAFGSAQALSEKDIQSVA
DHYATVEYFLKDGKYLVGNEPTIADISVWCTITNTFTYSPLDEAKYPKVKAWCKRMEQLPYAN
INIEGGKAFGQMIDSLRNRK

>PvGSTe6

MAPKLYASKVSPASRAVEMCAQAIGLKLDVVYFNLDGDHLKSDLAKINPKHTVPTLDDNGFY
LWDSHAIMIYLIEKYGRESNLYPADIKSRALVNQILYFDCGDLFANHRATVGPLFFENVKELTAK
ALANVSLNYPVVEQFLQSQNFVAGNELTIADFSIWCTITNASIYSTLDESKYPNIKAWRKRMEL
LPYASINIEGGKLFGNVLRSLLKK

>PvGSTo1

MKISYLKRYTSVLFRLASKDLKMATTHLTIGSEDPPKVEGLLRLYSMKFCPYAQRPLLVLKAKQ
IPHEVVYINLFQKPEWYMKIHPEGKVPALLDNSNVVVESIDICDYLDEKYPENPLYPAEPAAKN
KDKEVIKLIGPATGAFARCAFTDEIRTPESWVELFVSALQGLEDELKSRGTKFYGGNKPGMVD
YMLWPWAERSGCISIKLGYKLPFKDDDVPLLRRWKKDMSQQPVCRELFVSGERFWKIASCKL
TGIEPPYDEV

>PvGSTs1

MPEYRVLYFDGPGRAEPIRMILTYGGIKFEDVRLSKEDFLKLKPSLPLGQVPVLEIDGKQIPQSIA
ICRYVATLVKLSGNDAKENLRIDVAVDVLQDLQKKLFDYRSGKDEAERAALKEVVVNETVPFF
LKRIDKIAGENGGYIAINRMTWADILFINVYEGMVNILEKDIIVEYPNLAKIKSNVLAVPSIKEFI
KNRTERPGFYNLKKDL

>PvGSTs2

MAPIKVTYFNLTALGEPIRFILKYADIEFEDIRVGEDEWPKFKPTVPFGQLPIIEKDGKIVNQSVAI
CRYLAKQCKLNGKDDWEDLEIDATVDNITDMRTSEYGFYDKRLVFHYVVATLYSVCNKRISSQ
TVNCYSLKYETKLN

>PvGSTs3

MPEYKVIYFDFTGRAEPMRMILTYGGIKFEDVRIGREEFLKLKPTLPFGQLPALEIDGKIIPQSIAI
CRYLATITKLDGKDAKENLRIDVAVDSILDIQTKVNSYGMERDEEKKKSLLEPLKEAVSNILKV
WERKVAEHGGFFAINRMSWADILFICWYEGARSMLKNDILAQFPNLLKVKQNVLAVPSLQEWI
KNRPLLKFPDAYDLRQDL

>PvGSTs4

MIDWCLSRPWLFSSQIEMTESYKLHYFDFNGRAEPIRMLFAYGGIPYTDNRISIEEWPEIKPSMP
YKQLPVLEIDGKVIPQSIAICRYLANKVHLDGYNVKENFRIDVTVDTLTELLIKIIDYALEEDESR
RSSQVKKLLDETIPLYLTKFEERAKINGGYIALKRLSWAEIIFICLYEDATNFLEKDIIADYPNLIQ
VRKNVLAVPAIKKWIQTRPPCEMNTFYDLKSDL

>PvGSTs5

MAPAYKLTYFNVTALGEPIRFLFSYGDIEFDDVRVEKEQWPSMKSSMPFGQMPIIEHNGKVAHQ
SIAITRYLAKQVKLNGKTDWEDMEIDSAVDTVNDLRAKLAVYHYENDEVFKKVKAGPLFDEIL
PFYMEKLEAQAKQNNGYLACGRLTWADMYFVGLLGYLNVMAQKDIIVNYPTLISIKERVLGL
PKIKSWIDKRPQTPM

>PvGSTs6

MAPVYKLTYFNVTALAEPIRFLLSYGGIEFEDVRIDKDKQWPTIKPTVPFGQLPVLEFNGRLAH
QSIAITRYVAKLVKLNGKDDWEDLEIDSAVETINDLRNKIGNYRYESDEKIKQTKAGPLFAEILP
YYLGKLDAQVKQNNGHFACGRLTWADFYFVGLLKYFNFMTKRDIIEGYPNLVSLNERVLALP

AIKAWIEKRPVTEI
>PvGSTs7
MAPTYKFTYFNVTGLGEVTRLLFHYGGIEFEDVRIEEEQWPLLKSTMPYGQLPILEHNGKTYY
QSRAIARYVAKQVKLTGRNDLEDMEIDGTADVVADLFQKCVNYFYE
>PvGSTz1
MASMIEKPILYAFWASTCSWRVRIGELRYLLACKKETTYFSALNLKEIPHEIKPVDLVNGAHHT
EEFRTINPMEQVPALVVDGATIVESMSILQYIEETRPQKPLLPRDAIKRAKVREICEIIVAGIQPLQ
NLVVQERLSAEQKEGWVKFWIERGFTAVEKLLSISAGKYCVGDDITLADCVLIPQVSKSREFKV
ELEKFPGIERISKELAQHPAFVAAHPRSQSDCPPDLKYKD
>PvGSTs9
MIDTLEDLKQKLYSYGFETSEEIRKAQGEILKQMVPYVMELFEQRAKINGGYIALNRLTWMDI
NFLCTYESLRNILNTEVLTTYPNLAEVKKNVLKVPAIEKWIKNRSSTETLQIYNLKSEYSDIKKE
EL
UGT
>PverUGT1
MAKWVPQQQVLAHPKVILFITQCGIQSLEEAIFFQVPMVGVPFMWEQPSNAGVLERKMLGKR
VDIQNFTKNNFKRAILEVAKNPVYKSNIVELSKLSKDVEK
>PverUGT2
MPFIADQTMNIKRLQEFGVAEGIDYLTMTKEELKEKIIEVAENPKYKTNMKNLAQLWVDQPMK
SLDRAIWWIEYVIRYKGTAHLRSPTIEVSWIEYLLLDFIFLVIIIGLCLRYSKVIFTK
>PverUGT3
MFWYSALFISLIIPPSYPAKILCVFQFPSYSHQIVYQPLWKALSLRGHQVTVITPNPLKVTNLTNL
TEIDTSSVGNFLTFEDALQNSMSKEAPSTEIMEGFYRTSAKLFEGILGLPEVVKLYNDSTTKFDL
VITECLHPGMYSLAAKFRALAIGMSSLGVVVATHSSLGNPIHPVLFPDMNMAEHEQLNWRQK
LHSVYFSMWLLYYYNYVVLPESDTIARKYLGGDLPYLGDVGRDVSLVFVNTNPFIYTVRPNVP
TVITLGLMHLKFPEALSEDLQFILDESDNGVIYFSLGSNVKPSKLPKNMQSVMLNTISSLPYTVL
WKVDSELLDIEDLPKNVYIRKWFPQSDLLVHPNVKVFVTQGGLQSIEEAIVRGVPMVGLPFFG
DQAMNVRKISRAGIGIEVDPTTATVRDLRAAILEVAQNSQYRLKIQEVAKILVDQPMSGVEKAV
WWTEYVIRNNGTKILRSPTADSVWWQYFLLDIIGIVFVVIFICYKSIWWMSGVSR
>PverUGT4
MSVQHKPTLSKTVFRSAIDEVITNPSFKENVRRLATLALDQPMNGVDAVVWWTEYVIRNKGA
KHFRSVLGDLPLYQYFFLDVIAVTVVIVFIPLYSLWKLVTWLKKTFFG
>PverUGT5
MYNTYSMQVMWFCVGLFLHLRNTADSSRILAFTGFPSYSHQVIFNSIWRELSLRGHDVTVVTP
VPTRDPSLTNLTEIDVGFARKVLHDNNWVDKMAKDSFITTKVYYVLTIMNDLTEKEMQHEDF
QKLFKLPERHFDLVIVQACSPLVYALGAKFKAPIVVISSMPTLPNIQEALGVPTHPVLSPNYLINP
EGDMTLFAKIQSLFFEIIFRIIYKWIILPKSEAVAREFLGDIPPLGDIERNISLMFMNNNPFLHPIRA
NVPALIEMNQVHITSERKPLPKDIKEFLDSAPRGVVYFSLGSNVKSSSLPPKTRDVIIQALRELPY
KVLWKWETDTLPNQPSNVMARKWLQQQDILSHPNVKVFISQGGLQSIEEAMAYAIPMVGLPFI
SDQPANIKKITHLGIGLGLDPSTMSKEQFRNAIIEVASNSRFKKKIMEIRSILVDQPMKGVAKVV
WWMEYVIRHKGARHLHYPAADMSFYEYFMVDVFLFLFTCFLVFIYVIIKMLRLVRTMVNTRK
VKIN
>PverUGT6
MELKVLLLITLLGIPNCTESARILASIFSPSFSHQVAFRPLWRELAKRGHHLTLLTTNPMQDPHLE

NIVEIDLSGSYEIMEKYGR
>PverUGT7
MSTKLLFVWCVVLFLSHVAGAHRILGVFPLAMPSHYFLGNSLLKGLSDAGHDVTIVSVFHEKN
PPRNGTYQEIFLDGLYESQRGSEIFNSVLSVKKTQTNFIPFLWTYRMNNLGYFVCEETLKHPKM
QQLIRSDEKFDIVITSQFLMESLKGLAPHFHAHQVIFNNVMANSWMNHLVGNPSIPSFHPEIML
GLPEKMSFSERMYNSFYKLIQTLNYHLFYYPIHDELVKKYISNNLSLHDVLYNVSLVLSNSHPS
LTIIQPSVPVIKEIGGFHVSKPNVLPKDLQSYLDSAEEGVIYFSLGSYLKSEDLSTNTKSAILRAFA
KRKERVIWKWDDEVLPGQPSNVKLAKWLPQTDILAHPNIKLFITHGGYLSRTETVYHGVPVIA
LPILGDQRMNAKTCENDGYGISLDISTLTEESLTAAMEEIISNPKYIENAKIRSRIMRDRRQTPME
DAVYWIDYVVRYKGADHLKVPYLELSWYQFYLLDVGLVILCTILIPISILRKL
>PverUGT8
MRSVLLFMLFTHSACGYRILGVFPMSSKSHYILGGTLMRELAKAGHNITVVTVFHDDKIPGNS
TWREIFLDGLIEKQNGEYSNNDTLINIENQCQEFLASFRIHHMIFNSINFSFYYGFNNKSC
>PverUGT9
MHDPNLKNVLEIDLSGSHDVMERFRSSETMTSGSSSGRPMLLMQLEMLHVLNVVADWQLSQP
DVQQMIG
>PverUGT10
MRSIMYNFSMMFLNVHPAFYSRPMGPAFLTIGGGTHLTAPKALPKDFEEFIANAENGVIYFSLG
TNVNSERIDEKHRRIFLDTFRQLPQKVLWKFDLKDMPNIPSNVRIVDWAPQQDILRHPNVKVFI
TQGGLQSLEEAIFSHVPLVCIP
>PverUGT11
MSHASLSGPVPLQQNVKVIGGYHIPPVKPLPKYLKDFMDNTTEGVVIFSMGSNLKASHFEEDK
RNAIIKAFSKIKQKVLWKFEVNYTDISDNVKILNWIPQNDVLAHPNTVAFISHGGLLGTMEALS
HGVPIIGLPVYWDQVKNIADASRRGFALRLSYDDLNDQNFQGLLKEILTNPSFRENAKKQSRIL
HDQPVKQIDEAMFWIEYVIRHKGAPHLRTAALELKWYQLHSIDILLFIISIVLVSLLIINRIVNKFI
KNSRKVNIEKKKT
>PverUGT12
MVGLPFITDQPGNVRKLSDLGMGLGLDYSTMTKEQFRDAIIEVTENPRYKQKISEARSILVDQPI
KGVSKAVWWIEYVIRHKGTRHMRSLAADMSYYEYFMVDVILFFLACVFVIVYSVVKLLRLLR
NIVSSPKVKVN
>PverUGT13
MSRYRVTLFFGPKLLGLILLFCNAVSGANILITEDLASPSHQIWNYAIADALVAKGHNVTILGPK
TFPTEHKKFHFIPLEGLTEATTGSGVFLPEALMDLSLLGNIQLINDFSLFACNHSLQTKGLRRLID
YPADFKFDLIILDLTITQCFLPLIERFNSPPAVAISALILPPNLSELFGNSFPVAYVPYLLLKNKAGM
NFFERLQNFVYSKIDYYWYVLYTTPSMEQMSRHAFGENTLGLDTLGKKVSLLLCNLSPGFHYP
QPVTPNIIPVGGLHIDTKKKLPEDLQNIMDSAKDGVILFSLGSNVRSDSLGLEKRKNILKALSKF
KQTVLWKFESDLENLPKNVIIRKWVPQTEILAHPNLKLFITHGGGLSTIEAAFFGVPMVGIPFFV
DQHSNMELVEARELGLILNYKTLNSDTLFETVNNVLNNPKYSKNAKTISSIMRDQPQTPMERA
LFWIEFAMRNNDTNIYDPESRHLSAFVRSSLDVYAFFLFSFCAFAYFLMKVFVFLSSLVNSNQK
KIKTN
>PverUGT14
MSLRDSVFKMRSVTYLLVIHFMYLCSAEAARILAHFMIPSISHQTFYQPVWRELSSRGHEVTVI
TTDPLDDPSLTNLKEINVNATYEAIREMNVQDSYSRNNSVFHNFYSIMEDLFLGLLEYELGVAE
VQELMKLDENHFDLVIVEGLHPLIYGYGGKFKAPVVAITSIEMFLVHYDAFGNPTHPLTAPDWL

AFYENEPTIYEKFRVLFYNVWSRALYYWHIIPKADALAKKYFGKDIPPLLDLVHNTSLLLVNRN
PILHRPRANVPNIIEIGQLHLKDPKPLPKDLQQYLDNSTRGVVYFSLGTNVFLSTMNPSLQENIR
EALREIPYDVIWKFESDDMPGKPPNVLLRKWLPQQDLLRHPNIKAFVTQVGLQSLEESITSGVP
MVGIPIIGDQLLNVKVLKNTGTGIGLDIETLTKDSLKKAIEEVAGSNMYKEQVMKYKRLLQDQ
PMNDLEKVVWWIEYVIRNKGADHLRSPAARLSFYEYFLLDVVVYLTCASCSLVYVFRRLLRLC
SRFNNYKR
>PverUGT15
MYGKRLCMITASLAVINICTCANILMITMGGTKSHKIPFWELAKGLIPREHNITFISAFPADFLLP
GLEEITPAGLVFYVRNFTNWDLVGARMKGEDPVHPFDMVRYGYEACEVLLSDTETKDFLHQR
RKFDLLILDGAYPECALGLVHHFNAPFMYINTVGFYVGSLSLAGNPVPYSITPFLAMSVTDNMS
LLQKTQNTLWHVAANILHSVMVRFILQNVVRTHFGEDLPPLYDMSTNVSFILQNGHPTMTYPR
PYLPNVAEIACIHCKKSKPLPADLEEFIRGSGDAGFIYFSMGSSVKAVNMPIFLRKMLMTVFRKL
PQRVLWKYEADEEDMLDLPANVKLGKWLPQQDILGHPKLRAFVTHGGLLSMFETVYHGVPV
VSLPVFCDHDSNAAKAEVDGYALKLDLSTLSADKLLWAIKKVIHDPKYREEVKKRQYLLMDQ
SETPLQRAVYWTEYVLRHKGAKHLQSHSRHLGVIQYYILDVVALLIFGAVFIYFIVRFLLKLML
QHFLTSGIDLSKKNIKVE
>PverUGT16
MMKLLICVLCVQLSILSLVDSAKILAVFSFCAHSHFWLGFRLVQELAERGHDVTMINCFPQKNP
IKNLRDISVADTEPELRGKSSMIDE
>PverUGT17
MNTYTSIVILITIWISKNCDGLNILIIFPHMGKSHQLVFEPLFKQLALRGHNLTVISRFPQEHTLSN
RRDIDIGDGEKDTEFLKMGVWMGTKMSQFKFRFMVVEQSKFSCGDGLAKNNLLKFLKENNR
FDLILAEMFNTNCFMGIARLFGAPIIGLSSCHAPAWIHKFFASPQNPSYIPTHYSTFSDEMTFFER
VENTLSLVWDNFLYEYLISGPSNEFSKKYLGIDVLKGRDIMYNVSLLLSNTHFTLNLPKPNVPN
VIEVGGIHIEKQKKMPKNIEKYINDSSEGVVYMSMGSTLSGSSFPIAKRDAFLKAFSRIKQRVIW
KWETEMENKPKNVEIFRWTPQREILCHPNVKAFISHGGLLGTIEAVHCGIPVIAIPQLADQNMN
AKALEKLGTGVILLLDDITEENILEALQKVMSTKFRQNVKDLSERFRDRPMSPMDTAIYWIEYV
AKYKGAPHMKTAAVSMPAFQYLLIDVIAFVTLVTISFLYCLYKFVAFLFRHSRKQAKVKKS
>PverUGT18
MKYPSILLLISLWISTSCNGLNILVIFPHIGKSHQLVFEPLFKQLALKGHNLTVITRFPQNQVTTN
WRDIILGENDGTTEIFDMGVLTGTRLSQFLCRSELAVFGKNSCRDGLSNKNLQQFLKEKNHFDL
ILAELFNTNCFLGMAELFEAPIVGLTSCSAPTWIHRFFASPENPSYIPTHFSGFSDHMTFLQRVEN
TLLLGWDKFLYDFFISGPGNDNSKKYLGVDIMKGGDVMYNVSILLSNTHFTLNLPKPNVPNVI
EVGGIHIRKPRKIPEKIEKFINESSDGVIYMSMGSTLKGSSFPVPKKDEFLKAFSHIKQRIIWKWE
TEMENKPKNVETFRWTPQREILCHPNVKVFISHGGLLGTIEAVHCGIPVIAIPQFGDQPINSRALE
RVGGGIVLRLEDTTEETISKTLQKVLSPEFQKSAKDLSERFRDRPLSPMDTAIYWIEYVAKYKG
KPHMKTAAVSMPAYQYLLIDVIAFIALVIIILFYVSYKFGAFLLIKLRKQTKIKRS
>PverUGT19
MSPVHMAFAERFNIPFIGITPMDAPYRIHALMGNYIHPIAYPDHYLPVATPLDFRGRFTSTLFNW
LNWFLQYYWVYRRQTQIVRKHFGEHFKDLSEVEKDMSLLLVNV
>PverUGT20
MKYIQYMCIFLYVYSSECVRILCFFPIPSISHQIVFQQIWKELSLRGHHVTVITPNPLNDRMLSNL
TEIDVRISTYTHIYASTYLDHLNSQISLFSILPFYYDFFRSILIHELMQNEVLELINSNQTFDLLLLQ
VQMMMPLVYGFPAKFKAPVIALSSLEVFLHTHDTFGNPTHPIVSPDMMLTVEDNLSFMNKVES

FLYNIWYRMFYYWYVLPESDKTARKYFGEDIPYLGDIEKNTSLLLMNVNPVMHKIRPNVPNIIE
INQMHIRKKEPLPEDIQKYLDSSTRGAIYFSLGSNVQSVNLTTHTRNVIIGALADLPYNVIWKW
ESDYLPGKPDNLLLRKWLPQQDILGHPNVKVFVTQGGLQSIEEAITNEVPMVGLPFITDQPANV
RKIVKSEMGLGLDHRLMTREQLRDAILEVAENTKYKEKVKHYKAILTDQPLKGVDKAIWWIE
YVIRHKGAKHLRSAAADMSFYEYFMIDVILFLAVCIIISTYAIVKISLLIVRLTKKPKKKVKIN

CYP

>PverCYP6BS1

MPEKNINQLLPDNVPTVKILLSCIVCLLYAYFKWAYGYWKSMGVPQLNPTFPFGDMASVIFRK
QNMGDKIKEIYDKMKGQRYVGLYFFSRKAFLPLDPVLIKDILAKDFQYFYDRGIYYDEENDPL
SAHLFSIAGPKWKNLRAKLTPAYSPGKLKYMFDTIVRCGHEMTTILKEIAGETANGAVEIKEILA
RYTTDVIGCCAFGLECNCMRNPNAEFRAMGKRAFTQTIGDILKMIIIRSFPKIAKIFKIGVFSKD
VTSFFNKVVKETIEYREKNNIIRPDFLQLLIQLRRNGKIDEYDTDEKQVAPGTTLTMEEAAAQA
FIFFLAGFETTSTTISFALFEMALNAEIQEKARREVDKLLEIHGGLTYDCLMEMNYLETVIFETM
RKYPPAPVFLRKCTKTYRIPQTGVTIKEGQSVLIPCYGLHKDPEFFPEPELFDPDRFNEENKGKI
WDYTYMPFGDGPRNCIGMRFAMIQAKIALSLTLKNFKFGLNKKTILPLKMKTKGIILAPIGGLW
LDLKKI

>PverCYP4AA1

MGNKQVLVFHKISAMAYLEFLVKTVLSFFQFIVNYMRIVYLAFKLRGPPAVPFLGNVLLLKNEE
EMEAIGTNAHARYGPLMRIWISMLPVIFVYEPSHLRLVLGTNKYSEKNLFYSILHNFIGEGLITN
NGYKWKKNRKLIQPLFHVNILETFIEEFQKSADRFVERLEGFVDTNIKISSLVNDCVLDILHNSV
LGIPMDQDSPYRKGEVLLTTRMLKPWLLLEKVFKQTESAEKEKQQKRSLHEYTKRVLFQKRST
QSFSQKCLMDMLVEVVGNNKEFTDEDLINETVTFMLAGQDSVGAGLAFTLFYLAKYQDIQQK
VVEELKTIEYPNTMKGLNAMKYLEQVINETLRLAPSVPIMARVLKEEVVLDGKTFPSGTNLFIS
PFITHRLPHIFPNPMQFDPDRFSSENMEKMHPYAFLPFSLGPRKCIGYKFAYMEMKSVLSAVLK
RFEVSLKPGYERYQTVYRTTLRAKGGIWIQLKRRTPSS

>PverCYP6BQ1

MDIFSISYILLPLLSIVYIFSKWAHSYWKRQGVEYLDPEFFYGNSKELFQGKLAFGDSFAKIYNS
FKSRGLKFGGMYMFLKPVFMPVDLDLIKTILQKDFDHFRNHGMYINEKVDPLTGHLFNLEDEK
WRRLRAKLTPTFTSGKMKMMFQTMVACTSGLEKILTPHCELQDPIDIKDVVSRFTTDIIGSVAF
GIDCNSLENPDSEFRQFGRRIFHASFVRRMKNFLTMVIPKPLLIKTGLKQISSDIEDFFIKVVRDTI
NYRESNSITRKDFMQLMLQLKNRGKVSDDGEDATAPIGQTEGEFLTFNEIAAQCFVFFIAGFET
SATTMTFAMLELALNQHIQEKLREEIHDVLEETGGEMTYDAIMKMSYLDKVVNETLRMHPPA
AGTSRVCNTDFKIPGTEVVIEKGTDVHIPFQAIHMDPDHYPNPEVFDPERFNEENKAKRHNFAF
LPFGEGPRMCIGARFGLLQAKVGLVAILKKFKVTLDGKTKTPIRYSNSNFITTVKGGVWVQVS
VL

>PverCYP4BR

MISKPELLESLLTSNVHLTKSDGYDLFKPWLGDGLLVSVGRKWKSRRKMITPTFHFKILEKFMV
IFNKNFDLLIDILAEEVAKNGDKSIEITRYINLASLDAICETSFGTPLNAQINGNREYVKSVTEILE
IITLRFFSVWLRNPLLFRFSSLYPKYKHNLKVLHDFTNAVIVERKEASKIRKKGAQISDDGIKMK
AALLDMLLEATCDGEELSDEDIREEVDTFMFEGHDTSATAMCYVLYAISQNPDVQSKVYQELV
DIVGPDSKTELTLSQINNLKYLDIVVKEAMRIYTPVPIIERRLEEDWTIDGLTIPRDTNISIFIYGM
NRLPEVFPDPEKFDPERFLPENQAKRNAYSFIPFSAGPRNCIGQKYALYELKACVIKFLLKFDIM
EDASFQPEIGMSSVLKSRNGIKIKMRPRRMVE

>PverCYP6BQ4

MLDPSYPTIYLLGLFLALCLYLIHFYQASFQYWRRRKVPDLNPTIPFGNTLDLFLGKETFGSMF
QKAYLELKQRGLKHGGFYYFHKPVYMPVDPEIIKKILISDAHNFPDHGLYLSPKDPLSNHLFNM
EGGYWRSLRSKMPANFTSAKMRHMYGFMRELAEPYKQLLQQHAASGEAVDIKSLVTRFTTDII
YACAFGMESNTMRNENQELLKHGRLFFDNQWSLWRNSLVVTVPRSLLNKLNFRVFKKETEHY
IMDMYNSIRKHRKREGSTRNDLTDCLMRLTEKHEDELDFEGKEVLEPIDVENFAAQMFMFFAA
GFETSSSTQTFVLYELARNKNCQQKLRKEIHEVLERHHNELTYDAIMEMKYLEKVIDETLRIYP
VFPILPRVCKEDYPIPGTEVVLEKNSFVMISNMGIQRDPEYYPNPDKFDPENFSTERNATRPHVA
NVPFGEGPRICVGKRFGLLQTKVGVITIIKNFEVSLNEKTKQGFQFESKELILRKRGDVWLNLK
TIEQVPQ

>PverCYP314A1

MFEEIFYYLNSRYIVFIVIFIILGHRPKWLQVNKLVWLQKNVKDIPGPLSFPLLGTRWVFTFGGY
SFNKIHEFYVDMYKKYGPIMKEEALFNTPVISIFEKADIEKVLKSSGRFPIRPPTEAMAQYRKKR
PDRYASTGLVNEQGEVWHHLRTTLTSCLTKPKTIFDFLPQVDDIADDWCALIKQQRDRNNEVL
NLEDLMGRLGLETACTLVLGKRLGFLLPGGESEIALKLAEAVHQNFIAMRDTYYGLPLWKWFE
TSAYRSLVESEDTIYKLALDLIESAEDSTYDSVVFQSVINAKIDRREKTAAIVDFIAAGIHTLKNS
LVFLFYLIAKKEGTQQKILEDSSKSYLRACILESFRILPTASCLARITDTDLDLSGYKIKAGSVVL
CQTGIACRNNEYFDKANEFIPERWLDSEKSETSSNATFLVTPFGTGRRICPGKRFIEQVLPVIVES
TLKCFEIRVTKPMELQFEFLLSPKGPTTMTFEDRC

>PverCYP9Z2

MKAQVEIMDDFSRRFAEHHVNPSGDVTEIEMRDVTTRFCNDVSAKLSLGVSVDSFAEPDNDF
WQMCHRAVDTISMQKVLSFTVYLVAPRLVKFLKMKIVDWEADSFFRQVINETMKMREKEREN
GIVGSDLIHLLLEGRKKAQLNKSKISNSNYKTYNADWSNIEIIAQAFFFHFASCAPAPTLLSFMA
YEIAVNPEVQKKLRKEVRDIHENCQGKLTYEVFNAMKYMDMVVHGKLNEIPYTGCPKSTKSS
QE

>PverCYP348A1

MSYCSPASNSFLYYIDENLLFTAIASITFATVAVFWYILRSYTFFDKLHVVGRAVRPKAPFGNIKD
VVFRKTSLYIYLWKYYNKFKRQGHKFGGFYLFLKPGILLVNPALTYRILTDEKLFVDVYRKND
VEECKKITRLFNSDVLHNLILKCNTFKNQFIHDIGDKSNFSSVVHDYLYETTSTVFGFKLEEKLK
KIVNNIIEEKTASNLSHYFHLVYPCFVKKQTRKELKSYIQAVMNSRKKNDIREDDILQNYIDFIN
DNDDDLETLILDELLDVFIVNIYQTLNTVLFCLYEVASNSDIQQDLIGEIRRFNKANSSINLNNLH
HLIYLEAVVKGRLSS

>PverCYP303A1

MYLLLIVSFFCLITYLLTRKPPQFPPGPLWLPFFGSALQLASLQKRTGHFVLATAELARKYGPVL
GLKIGCEIIVVVHGTKANKEFLSSDDLSGRPIGDFFDLRTWGKRRGLLVVDGKFWVDQRRFFL
KHLRDFGFGTRNMSRLIEDEARELVQHIFRTMHHDDSAVMNIPTLFNVHVLNTLWKMLAGVR
YDAEDKRMLELQEILNELFRSVSMVGTTFSYFPILRYLMPETSGYKLYDNSHKRIWEFLRKEID
HHKRTRETGAPRDVIDVYLDVLETSEGASESFSEDQLLATCMDMFMAGSETTSNSLSFCFLYL
MLNPDVQAKAQGEIDAVVPKDRMPTLSDRPRLPYVECVVMESLRMFAGRAFTVPHRALKDT
YLSGYLIPKDVIVIASLYGSMLEEGCDFEEPERFKPERFLKEGKISLSDSFLPFGFGRHRCLGESL
ARANVFVFVATLLQKFTFSCAPGYPLTGEWIDGITPGLKPFKARVRPRY

>PverCYP18A1

MFADNMITVWNFTVQECCSTGVLVVFLTTLFLVRGIQLFLQSRALPPGPWGLPIIGSLPFMKGD
LHLYYLELTKKYGSTISTRLGSQLIVVLSDYKLIRDTFRREEFTGRPNSEITSILDGYGVINIAGKL
WKDQRRFLHDGLRHFGMSYLSSRKAQMENRIMREVEEFLMVLKARSEAPVDLNPVFAVSISN

VICDVAMSVRFSHNDSRFIRFMDLIDEGFKLFGSLEAALFIPVLKYLPGHNSTRRKISQNRDEM
AQFFQETIDEHRETFDPSHMRDILDTYLFQIQKADEEGTGHHLFEGKNHDRQMQQIMGDLFSA
GMETIKSTLQWAVLFMMHHPDKMRAVQEELDQVVGRQRLPKLEDMGYLPVTESTIYEVLRIS
SIVPMGTTHAPIRDLKMNGFHLPRHAQIVPLLHAVHMDPTLWREPEKFDPSRFVNVEGKVTKP
EFFLPFGVGRRMCLGEVLARMELFMFFAAFAHSFDVSVPEGHTLPSLKGVAGVTISPKPFRVRL
RARRMDWDQAEGGLRTAGSH
>PverCYP4BN1
MSYVQTYGPIIKVYDGPMSVVVFVTDEKFLEYILSSTKIIEKSDQYNLFHNWLGAGLLISTGMI
EKIYIFL
>PverCYP4G7
MNCIVDLSILELEQCDTFERRKMSQPQVVPLGGDSTLLSSLIVSVVGVTVVIALYEIWFQSLRYV
RLAKNIPSPRAIPLLGHAYLALGKSPANVFKLAISLYRKLKSDVVKVYLGPKLVVGIVSAEDAEI
ILGSTVHLEKSPEYGLFEPWLGNGLLISKGEKWRSHRKMIAPTFHTSILKSFLPVFNKNANQLV
EAFRKEKGKEFDVHDYMSGATVDTLLETAMGVTKTSEDDTGFDYAMAVMNMCNILHQRHYK
IWLRFEPLFRFTKMSLQQGSLLNTIHTLTRRVIKRKKENYFDRKSKGELNQYENVVDSGKFDER
HGLFEETKNIMTNYIRDDLDENDENDVGEKKRLAFLDFMIEASHSKGIHITDEEIKEEVDTIMFE
GHDTTAAASSFVLCLLGIHKNIQQRVYEEMKEIFQDDMNRPITFNDTLQMKYLERVMMESLR
MYPPVPIIARKVNEDVKLASGDYTIPSGTTVIVGQYLIHRNPKYYENPEKFDPDNFLPEKCQQR
PYYAFVPFSAGPRSCVGRKYAMLKLKVLLAGVLRKFVIHSNTTEKDFNLQGDIILKREEGFKIT
VSERA
>PverCYP349A5
MIMPSFGQKVLDSYVEKFNERAEILREQLQKHIGETNFGLREKLFKYAVDVVCETLMGVTVDF
QTSDSDAGDDLNRILEILTIKIYSVWYHIDIIWKLSPLYRELSYLSKKIRKHHFMSKIREKMNEQR
NRISKEETQNEFQKKLPFLDWILQITDFNETEMIDEIDTFMAAVSFSLR
>PverCYP9Z3
MVDILAAASNFICFLVNMWTLLLLLTTLLILLYYYTIKPLYYWRNRGVKQEPFLTALIDNYFMIF
KRQSAAHALEKMCQSFPNTRYFGLYQFNVPFLMVTDPELIKQITIKDFDYFVDHRAFVPADIDP
LWGKNLFALSGAEWRQMRPTLSPSFTSSKMKGMFCLMKACAETFVRHFQGRDEEIVTVEIKD
VTSRYCNDVIATTSFGVEVDSLERPDNEFYRMGKKATNFQAVWKQVRLMGYSACPTLYRFLG
LKLFEDDVATFFRSVIDETVKMREEKLIVRPDMIHLLMEARKGVKYNDEKGVQSTVEESSLGS
FSNEDITAQALVFFFAGFDSVSTLMCFLAYELAVNSDVQDKLREEIKDTLRQCNNKLTYDALVN
MKYMDMVVSEVLRKWPVNMATDRVCTKPYTIPPKSPREVPVHLDMGTNVFIPQFTIQRDPEFF
PEPERFDPQRFDEANRAGIAPGSYLPFGLGPRNCIGSRFALLETKVLFFYLLGGFEVVPVEMSIIP
IVLAKSMFYLGCQGGFWFGFKKIK
>PverCYP305A1
MPIVGNGPLLRKLSKSLGGQHLAFEKLGKDYNTEVLGLKLGGEYVVVATSHQTVKALQLSEE
YDGRPKNFFIKLRSAFTEKGITGTDGEFWRIQRNFVKVHLRKLGLGKKTMEFKVQEEVSEILNL
LDSKKGEPIQMNTILSISTLNVLWAFTSGSRLKHDDPQLTELLYLLTIRSDAFDIAGGLLNQFPWL
RLIAPEKSGYNLLVNINEGLRGLIKKTIEEHHENWSDGRDDDLIFSFITEMKKDEGKETTFTDDQ
LIMVCLDLFLAGSMTTSSTINFAMMMMIEHPDIQNRVRSCLLEEFGKTGEINYSDKNRVPYVEA
VILEVERLLLVVPVSGPRRVARDTELGGYRIPKNTTVLISLYSSHSNEQFWKDPKVFRPERFLDE
NGKLSVPDRFMPFGLGKRRCVGEILAKTAIFSFFCGIMRSYEITLAANSKISTGKLITGMTSRAG
EFESVFTKTSNQ
>PverCYP302A2

MLSPNHIMLSKSIKGCCRSKNLYYCLNHFSFEQFRRQLSFSSIPGPRSLPGIGTLYKYLPIIGEYKF
DELHSNGFKKYKDYGPVVREEIIPGVNVVWLFDPEDIETMFRHEGRYPSRRSHLALEKFRLDK
PDVYKSGGLLPTNGPEWLRIRKILQKGLSSPHAVKQYLTCTNDIIDEWIMVIEEISQQPDIDYLPE
LSRLFLELTGKTTLDERFDSFSEIERKKDSRTSKLIDAALTTNSCILKTDNGPHLWKKFDTPLYK
ALRKSQEFMEKTAINILLSKIVSFSGKNPTTLLENYLSSPDLDVKDVIGVMCDFLLAGIDTTTYT
MSFVLHYIATHPKVQERLKQESMYLLPTHDSPVTEDVFNAAEYTKAVLKESLRLRPISIGTGRIL
NNDAKFSEYSVPAGTVVVSQNQVSCRLEKYFPRPNEFIPERWLKTETAFTQPHPFLLLPFGHGP
RACIARRLAEQNMIVLLLKLSRNFRIGWDGGALDSKSYLINKPDSAILLSFCKRTA
>PverCYP49A1
MWSLREEYGKIVKVGGLVGHPDLLFVFDVNDIEKVFRREEGMPHRPSMPSLHYYKQQLQKDF
FSGNEGVIGVHGPKWDAFRKQVQRILLPPATAKKYIEPLDIIATDFLTRMEAMLDDNKELPENF
LSEIYKWALESVARVSLNTRLGCLEPNLPENSESQRIINSINTFFWNVAEVELKFPVWRIYKNKA
FRKYIDALEDFRTLCLKYINQSMEGMKDRNYENIKEEDISIVERILIKTENPKLAAVLALDLLLV
GVDTTSIAAASTIYQLSQNPEKQEKLFEELKSILPNQNSKINIEVQENMPYLKACIKETLRMYPV
IIGNGRSLQSDTVLAGFKVPKGVNLYLHILHIN
>PverCYP4BR1
MRKLYQEYKPRFVIYTGPQVRVVHSLPSHIQFIVNSNAHTTKSDNFDVLKGWIGEGLATSKGD
KWRQRRKIINQSFNTNLMERYLQVFNSSSNSLVAALANEVGNDFVNTLEYTNASALEIACENL
MGLKINPSKNPQEQDYIEKTRMMLRIVGVRFFSFWQRYESLFLLFSSEKDVYLSYAKSLKAFTI
DVIKKRSSMFLKQRKTPEKKDESGIKKKAAFMDIMLAEHFEGRMSYDDIREEIDTFMAAGYDT
SAGLVAFILYELARQPESQQALFEEIMRIAPEGDFTLQQVNDLEYLECVIKEGLRMYPQVPYME
RQITEEFELDGIKYPVGTVFSIPIIDMQNDPELFPDPETFDPSRFLPENAEKIPKYNYLPFSAGPRI
CIGYKYAMVSAKVSVAKCIREFEVLPVPGFELELEFQITLKSRTGMLVKLKKRVY
>PverCYP9Z4
MILYLLAIVTFLTFIFHRLLSPLNYWSKQGIKQRNPWGAFIFNWFIFLKRQNVVHFVENLYTEFP
SERYSGCYQFNKPALVLRDPDLIRDVTIKKFDHFTDRRTYVPEEADPLWGKNLFALKGAQWRN
MRSTLSPTFTGSKLKGVFVLMQSCARNFVDHFAEKQEKLIDVDVKDVFTRFCNDVIATTAFGV
EVDSLKQPENEFYVMGRKATNFSTYKVVVKFMGFLIAPRLYKLLRIKLFDDDVSAFFKTLMDD
AILLREQKKVYRPDVIQLLLEARNGESNSEASDNQSTTSRAPTITNEDITAQALVFFFAGFESLST
LMTFAAYELAINPEVQDKLRREIQKTSEKCSGDLTYDALVNMKYLDMVISETLRKWPSQVAAE
RICTEPCVLEEAPGLKLAVGDVVIFPTYGLHHDPVYFPRPELFDPERFNEANRAGIRPYSFQPFG
LGPRNCIGSRFAVLEAKILFFYLLGSFEIVPVEKSVVPVVLDKKSMVLAAEGGMWLGLKRLGSS
>PverCYP352A1
MYVNQVLFCWCSGLVLFVLLVSTEMPIFLILLLIIVFLYLQFYWSRRHLYAAAARINGPKGYPLI
GNAHLFIGDTADFFQKINSIFSHVDREPYKLWLGPYLLISLKNPIHLEKIMSSSKFAQKHELYVFL
ESFVGEGLISSSGIDPKYKTHRRIIQPLMDLKFVSNTISIIQEQTDICMKILQNHVDRSSFDIHDIII
NCSIDIMGEIILGQNINSQRNGPTKFCKALEEMYDLAYARMTKVWLHPDFIYNRLPLKRKSDAV
HRDLIDFVQKALIDSRRRRKIMKDDIGEKFTPTIDRLTEIIENNHNVMNDEDLVHHLITLFAAAD
DTFAIIASFSALCFGMYQEYQLKAVEEIRAIIGEEPRQMSMEDLYKLQYLDMCIKDVMRLFTIAP
YILRRNLENFQLDKWVLPKGSAIVIPIHHLHRDPAHWQNPDHFHPDHFLPEAIRKRHVYAYLPF
SSGPRGCIGKIMANVLLKVFLVNLLQKFEIHADGKVPDIALRCDISVRPKRGYICGLRKRKWTN
T
>PverCYP301A1
MTKKISNLMKSIIRRRSQTAFAVSETLCPAHAPDVQNPKPYAEVPGPKAIPVLGNTWRLMPIVG

QYDISDMARVSHLLYQDYGKIVKLTNLVGRPDLLFLYDANEIEKVYRQEGPTPFRPSMPCLVKY
KSEVRKDFFGVEAGVVGVHGTPWKTFRRKVQKPILQLHTVRKYIEPIEAVTQDFIQRMLEMKD
ENHEMPADFDNEIHKWALECIGRVSLDVRLGCLDANLPADSEPQKIINAAKYALRNIALLELRF
PFWRFFPTSIWTNYVKNMDYFIEICTKYIDEAMTRLKTKTVKDEKDLSLVERILASEPNPKTATI
LALDLILVGIDTISMAVCSILYQLATRPDQQEKIYRELKRVMPDPNEPLDADKLDELVFLKAFVK
EVFRIYSTVIGNGRTLQEDTVLLGYKIPKGVQVVFPTVVTGSMGEYVPEAEKFRPERWMKGKD
EKLKIHPFASLPYGYGARMCLGRRFADLEIQVLLAKLIRTYKLEYNHEPLEYKVSFMYAPDGEL
KFKMTKRKE

>PverCYP4Q1

MILPIWLEILVGGVGAFFLLKYILEWKRQIELMDRLPGPKSLPILKNILDIHCEEDVFWRKLRKW
SKDYGPLYNIAALHFPSVNVSGPEEFEVIASTMKNIEKGQVYLFLRRWLGQGLLTSTGSQWQIR
RKILTPAFHFSILQQFVGVFNKETEKLVEVLKKETSKSWTNTLPLISEFTLYTISETSMGCKLDLQ
NQEDKKYVSSIYTIGKMLFQRMNRPWLYSDLIYYNLTPTGFKERTITNIMHNFTDNIISLKLKDF
KKFEVPQEEYNYSQKKKLAFLDLLLNAKMGEGIIDDKGIRDEVNTFMFEGHDTTSTSICFTLM
LLANHKDYQEKVYEEVISVLGPSKTSTPSITELNDLKLMERVIKESLRLYPSVPFIARILEDDVVI
NGYLLPRNVPVNVHIFDIHRNEKYWPDPDKFDPDRFLPEVSAKRHPYAYVPFSAGPRNCIGQKF
AMLELKAVLCGILKNFQLDPIDTPDTVTLVPDMILRTQNQSLKVKFKLRNA

>PverCYP347A1

MGFFLYELASNPEYQEKLRIEIMDSYKKHGNKFTYESINDMEYLEACFNESMRKNSILHHLVK
VCTKSYTYTPVHPDYKNKSVTVETGIPIIIPLTGLHSDPKYWKSPEKFDPDRFLVKENIRKFTYL
PFGEGPRICIGI

>PverCYP6EF1

MASYIDFLTTLAVLFAITIAIYGYFMHSYRYWKKKKVPYLEPVFPKGNNRELSTKTVTVNLNV
MDFYDEIKSKGWKYAGIYLVVRPALVVIDPEHIKNILVKDFEHFVDRGFYHNEKDNPISAHLFA
LDTIKWREMRVRLSPTFTTGKMKMMFPMILEVSHYMMEAIEKQVDDCHDIDIKDFLARFTTD
VIGTCAFGIECNSFKNPDAEFRRVGQQIFQDDTFIGQVKKIIITNAPELALKMGIGSIRTEISDFFH
NSIESVIKYREENNARRSDFLQILIDLMNSTKDTDNSFTIDQLVSQVFLFFIAGFDTSSSAMSFAL
YELAMQPDLQEKLRNEIHAVLEKDDGNLTYQNINEMHYLQQIIDETLRMYPSVPLLQRRCVKQ
YTLRNTDCVIDKGTTIIIPIINIQRDPEYFSDPLKFDPDRFSSENKKQRNPILHIPFGEGPRNCIGSR
FGTLQTKIALVQILRKYKLSISPSTKMPLTSQITHGHPMDIQWTSFMNFMDILWTSNLYPKDVL
WTSDGRPKRSNLYIHRISSKDIHKISFNDIHKISFKDIHKISFTDIP

>PverCYP9AD1

MMTEVLLVVLTLVVAYWFCVKPSFYWTNRGVKQRLQVPLLGENWRTIMKLECFADMVDRIY
KEFPNERYNGIFQCCTPILLLRDPDLIKQITVKDFDHFVDHRSIVPENTDPIWANNLFALTGQKW
RDLRPVLSPSFTGKKMKNMFLLMLECAQNFTQNFLDKKLGVVEIELKDTFSRYASDIIATTAFG
ITVDSVNDPENEFYRMGLQAPFNGLKFIITFFLPNFLIKLFNISLVSSEVRSFFINLVEDTIKIREEK
NIVRNDMIHLLMQAQKGKLKYEEDKDLVDSFATAVESDIGKQVRSRMELTNMDIASQAFVFLL
GGFDTSTSLMCFLGYELAVNQDIQARLREEVQDTLIKCNGTLTYDALLKMKYLDMVLSETLR
KWTGGLVIDRVCTKPYTIEPKLPGEVPIHIKEGEILWIPLVGIHKDPQYFPDPERFDPERFSDANR
GNIEPYTYIPFGSGPRNCIGSRFGILETKTLFFHVLAKFEIVPVCRSQIPVKLSKNAVTLLAEKGF
WFGLKAL

>PverCYP6BR1

MVQGERDNTLFQAQYMNLFYNKIRQTDMLTEILILLVSLGISFALFVKQRYGYWKRRNVPYLE
PTFPIGNNTSFLPRGISLGIISKKFYDEFKTRGVRVGGVFLGMDPQLVLTDPEHVRTILIKDFNSFT

DRGIYKTESDPVTVTIFSQEGQDWRIIRNKFTSIFTTAKMKMMYDTLKACSTNMISTLDNSAEK
NLDVDVLEIMAKFTTDVVGSVIFGVDCNCFKNPDAEFRRIGKHLFDEFTLMDLIKLFSTIFFPN
MAGLLGISNIQPEVSKYFSGIIRDTVEYREKNNVNRRDFLDLLINLKNTNQQITVDELIGQAFIFF
AAGFETSSTTMALTLYELAQNQKSQDKLRQEVKTCMEKDNNDLTYETVMEMKYLDQAVEET
LRLWPPVTTLTRVCVQDYTYPGGDFSIKKGETVIIPLLGLHRDPEYFPEPLKWNPDRFADKIEKS
IAYFPFGVGPRNCIGMRFGLMQVKLGLAILLSRYRVYPGPTTESSLDIDPDTFVLHTTKRIYLKF
EKI
>PverCYP348A6
MKETLRKYPPAPHLSRRCQNDYTLPGTNILIEKGMAVYLPIYAIHMDPKNYPDPHTFNPNRFSK
ENIEDVKACSWLPFSVGPRNCLGERFATLNFKSAIANILYNYEIELG
>PverCYP6BQ3
MKDVLGEYYYFQARFKTDNISSCAFGLECNSLKNPDNPFRKYGYRAFQFAGWNAVQLIISVRP
RDISGFAISIRRSLRSSLVLCSHDVL
>PverCYP349A4
MYFILINVIIAFLLLRYLWKKRQLYILSWNVPGPFSLPLVGYVYTVTRLGVDDTVRLIEKSILSYP
SLARVWFGPFLVYFTTKPKYIQKILNNVFERPYLLRLGRFPGRKGLLASRVNVWKIHRKAMMP
AFNQKILNSFVGTFNEKAQILCKQLEDYEDTRGVDFFDPLFKCTLDIVCETTMGIEMNKQTTHS
EYGKSIERLVELLSIRIFNLFYHIDFFWRLSSAGRSEDEPYTILRKFTTKVLKKKIEDHNESKKTL
TVDPDRRLAFLDSMLEMGKFTEEELLDEVDTIIGAGSDTSAKTLEFMLITLGMHQDIQERVFQE
VMQIIGPDRNVDPKDIWQMKYTERVFKETLRLFPTGPVTARTLEDDLELDAEYTLPKGSMVFIF
LSTVHRNPDYWPDPLKFDPDRFLPENVAKRHPYAYLPFSNGPRNCIGEFHSQFSALLPEDISSKK
NKWIEYSQFSHNSMNPIYRSSFRHDEYNDYHGTYDKEVQILHGA
>PverCYP347A5
MFLMLMIIVSLLILIYFLVCAKLNYWKKREIPGPYPYPIIGNFGKLIIGKRGLSQIYNDIYKEYEG
YPFVGIYKFTHPVLLIRDPDLIKCIMVKNFSSFNKNDIFIYEDIDPVYSKTVFATDGSKWKTKKA
EMARCLSPSKIKYMYSQMEKNCLNMVHYIKQQLEDSENPDIFIIFKSLIFNNITACALGVEGPCF
EEISSGMRGMVDACVIAEGFFDRLFSLVWMNLPTLSKLLELSAISPELADSTLSTMKSSMKYRE
DNNLVVNDYLHAAMEADGTDHDKAAPIASLFIDGYLAIVNAMSFLVYELASNTHIQDELRAEI
NECYKSNDGNFDYDDTMKLEYLDACIHETLRKHSIVQVIGKICTEKFTYTPTNPEYKKIAVTIEP
GTSIMIAPFAIQRDPKFFPDPEKFVPERFMVENSINKFSYLGFGMGPRKCPGSRFAITQMKIGIAH
LIKNFRVIWKAKVPLEYNPWHAILCIRDGLDIELQRIEHR
>PverCYP347A3
MTSCFTSGKMKGLFPVLERNVDKMMSYMNEQMKTSSCFEAKEIFVRFMLDNVAACALGIEG
KCFDEPYSEVRKMADHFFFSKGITSLKFILIFNLPPLSKFLRLKVVSRDVEDKLLDIVKTTLQYR
KENNIVINDILDSIAQISTTQNVTELDITSHVATFFTNAFETSSLTMSFLIYELAQNPEKQEKLRKE
IKESYEKNNNSFTYESIQDMEYLDACFKENLRKHATVPLMPKICTETYKYVPTNPEFKPIEVTL
QPGTSIIIPSTAIHHDPKYYENPDDFVPERFLDKENTNKFTYLAFGGGPRMCIGTRFALSQIKIGV
AHLIKNYEFTVNPKARVPVKYDPFYFLLTASGGLWVDVRKVNP
>PverCYP346A1
MDFLQMMMQLRSRGEVPEREEHIVPTSRSRKEFLSFNELVAQCFMFFVAGFETSASAMTFTVL
ELARNQEVQVKLREEIEGAIAEDGELTYA
>PverCYP349A2
MPAFNQRILESFVEIFADQSEIFADQLRKNVGRKDLDLFKLISSCTLDIICETAMGVKMKHQTTD
CDFGHNMDRIMEIVIYRIFHIWNHSKIARIFFPINWEMRKCMKKFKDVTSSVVKLKMEEYQAS

KNTVVANTEEEEPKKRLAFLDLILESSNFTEEELIEEVEIFMIAGTDTTASALCCILTVLALHQDV
QQKVFEEIIEITGLDRRVQPADLPKMKYTERVIKESLRLFPVASFFLRKVGGDIDMGEFIVPEGSS
VFFGSVHIHRNPKYWPEPLKFDPDRFLPEEVAKRHPCTYVPFSFGPRNCIGIRYAMMAMKTILA
STLRRFKVFTLHKSIKDIELKSNIVLRMKDGPQVWIESR
>PverCYP9Y1
MMLLITLFCLTVYLLYTKFIKPVLYWRSKNIFHTKPWGRFLKVFFSERSFYESIIEAYRLFPTRRY
YGSYQFLNPSLFVRDIELIKKITVKDFEYFPDHFSFVNRKSDPILGQNLFSLEGEKWRDMRSTLS
PAFTSSKMKSMYLLMKETANNFVDYIHNQSGDIIELEMKNAFSKYANDVIATCAFGVHCDSLK
NDGNEFFVMGSRISQPKGFAIFRGILAAFFPYIFEFFRIPIFPRYITNFFRTLVIETISLREKNQINRP
DMIHLLMEARQGRLNHELEKNQEITTEFATVEEIIDDKTSKTVLNLTDDLITAQALVFFIAGFDT
SSTLLSFMSYHLAVDTSIQLRLQREIDDAIIAGNGTIAYEALLKMKYLDQVISETLRKYPAGFILT
RLCVRDYHIPALNADEVDFVLNKGTLVSIPVAGIHMDSEYFPEPDRFDPDRFSDENRNKIVPGS
YMPFGSGPRNCIGQYVLPNIE
>PverCYP6BJ1
MGQVLDETMRKYPALPILTRRCVEDYKIPNEDVIIEKGTKVIIPVRGIHYDEEYYTSPEVFDPER
FSDENKKTRHSYCHIPFGEGPRICIGLRFGIMQAKV
>PverCYP306A2
MDLDGVSNEKLFVNNGIEFQELALKMNSSRGDAYMECFTSVIAFLTILILYQIWLGWNRPPGP
WNLPFLGYLPWIDHQAPYKTLSKLAKTYGSIYGLWMGSVYTVVLTDVALIKMLFSLDSTTGR
APLYLTHGIMGGYGLISAEGELWKEHRKFARRTLKQMSTRTITEGGTNKIENLLTEQVTELISVI
FPLLNTFLVTFE
>PverCYP6BQ2
MMFHTMVDCTKELKIILDEQATSKEPIDIKDLLGRLTTDTIGSVAFGVDCNSLKNPNSEFRRYG
KMAFDGTPATRVARVVANFLPKWLFAAAGFKIMDKGAEAFFSNLVRDTTSYRESNNVFRKDFL
HLLIQLRNFGKVREDGVLIDEGATDNRQMTFNEMAAQCYVFFIAGFETSASTMTFALLELAQH
QDIQDRLRENIDDVLRKHNGELTYEAVMEMDYLDKVVQETLRKHPPGVILPRECNKTYKIPDT
DVVIEEGTRVFIPAYAIHKDPEYFPEPEKFDPERFSDENRTWPPIAYMPFGLGPRACIGSRFGKMQ
TKVGLLSIISKFRVTLNEKTRFPIKYGAAFVLTVKDGVWLNVEKI
>PverCYP307A1
MLAIVSLVLVLLVCWLSRRSRKDVSMGIPPGPKPLPIIGSLHLLGQHETPFEAFTALSKVYGDIFS
INLGSTPCVVVNNFSLIKEVLITKGGDFGGR
>PverCYP306A3
MEWYVYLLASLMAALLATYLVRAARDLPPGPWGLPLLGYLPWIDPSAPYRTLTALAARYGRV
YALRMGGVYAVVVTDVELIKRLLASDATTGRAPLYLTHGIMQGYGLICAEGELWREHRRFVGK
CLRRLGGDAGGLASLIAEHVGG
>PverCYP306A1
MNAIVLGRVWGRDDEEWLWLQQLQEEGTKSIGVAGPLNFLPWLRFLPSYRRTMRFILKGKRD
THELYRRIIAEQTGGRDNIVQAFLLEKSKRSPADARRFYNDQQFHHLLADLFGAGLDTTLTTLG
WYLLYMAAAPAAQEAVRAEMGDGPPSLEDAPRLPLLRASICEAQRIRSVVPLGIPHGASRELRV
GGYRVPEGAMIVPLQWAVHMDEAAWEEPERFDPWRFLDEEGRLRRPRNFMPFQSGKRMCVG
DELARTLLFAFAAAILHKFHLSLEEGSDVDFRGECGITLTPKPHKIVFTKVK
>PverCYP345A2
MLLTSSWIVDFLVLSATVAVFLYKYSTRKFDYWKKRGVYYPKPVPFFGNIADVSFFRICIGDWL
KKIYDSTDKPYFGIFVFDEPHLIIKSPELIKQILIKDFSYFSDRTVAAPKHNKIISNLMFFQKNPDW

KDNRTKLTPVFTSGKLKMMFPLIQDVGVNLLNYLEKNEGELEAKEVFAKHSTDVIAKCFFGIN
AHCFDNEDAMFRKLGKNVFDFTWRNGFVQTSYFFRKNWVDFFKMDFVEKWVLDWFNVAIS
GTIRARKTDSARKNDLIDILNELKNDDKYSSDTKLETEKLIGPALQFFLAGFETTSSTIAFTLYEL
CLNKNIQNKLRSEVMSSIKKYGSITYENLMEMKYMDMCVKETLRKYPVLPFLDRTCNEDYRL
PGTDLVIEKGFPVYIPMFGLHLDEKYFPGPNKYDPERFQDKNFNKEGLVYIPFGDGPRNCIGER
FGMMSTKATLAYVLAKFEVEKCAKTPDPIVFEPKSFVLQSKVGMPIVFNRIIPTPA
>PverCYP4BN7
MRICLPPHWTVVVITDKDVTKRIFEETIDKSTDYDYFKPWLGTGLLTSKGNKWKQRRRLLAPC
FGSLELMKGFINVFENLGIDLVAKLEKEVGNTNLDLIPLMKRYTLDVLCETSMGVSLTSDGELY
RESLESLCGVLVNRLKFPVKRIDSLFRFTKDYRKQQQCIKVIDSFFDEIFEKKLAALNEGKMNT
GKRLAFLDLLMAFHKTGELTKEDIRAEVNTIMFGGHDTTAAGLAFTLYSLANNPHVQEKVLEE
QMNIFGNLKHDNQQTFENIQQMKYLELVIYEALRLYPPIPVIARQLPNDINLGDIVLPKDLNVIL
FIYGSHYDPKYFHDPEKFIPERFESGEVLPFTYLPYSMGQRNCIGKKFGLIEMKSCLSKIVRHFK
LSPSIPLSEPKLVHELTLTSLNGIKISIERRD
>PverCYP307B1
MHLEEDPNMNWEHIIFELEDFIGGHSAIGNLVMITLAAIVQHPEVGESIRKEIDAITGNTRIPNLF
DRAAMPYTESVLWETLRRASSPIVPHVASVDTKIFNYSIPKGTLVFINNYELNVGKEYWNKPEE
FLPERFLSVAGNITKPKHFIPFSTGKRTCIGQRLVQCFSFVILTTLLQKFDISGGEDVVAKPGCVAV
PPDCFKLILTERKPPVITSE
>PverCYP4BN8
MDPYFVVLLGITGVVFFLYRRHNHLSWIPKPQCHFIFGHYELFQDTTGVLEKIKEMTIKNGGMI
RIHFPPMKPGILVTNPGILRCILKSDQALSKSKFYNFLKPLLGEGLLTINGDYWKGHRRLMNPSF
GRIPLLRNYILHFESIGDVLIESFVKESNNEHINLIPLMRVYTMNVICETMMGLSKNSPENNNFQ
YTRAIETIFKIVINRMKCLKRFNLIFKLTEDHKVLSDNLKKVDDFFASIMERKCTEVALEDTPHS
SFLDILLQFLRNGSGITEENVREEVNTMILGGHDTTSTALSFILYALAHHPDVQEKVYQEQTHIF
SVDDSKPQVSYDNLQDMKYLELVIKESLRLYPPIPVIARELTGDVVLDGGQVLPKGLNVGIFIY
GCHRDPKYFPNPEVFDPNRFMTDNILPFSHLPFSGGPRNCIGIKFAMLQMKSCISKIVRNFQLIPL
EAEEELILVPGVTLKPKNDLRICVRSRLGKHE
>PverCYP4BN2
MLTPTFHFSILENFIEVFNRQGDVFIKKLEGEIGKSFDIYPYVSRFALDVICEAAMGINLNSQEQE
SDEYTKAVKSTCGIIIKRSFSALSPSLYPFTIDHF
>PverCYP4Q2
MVYRVVRPWLYPSLIYNLTKKSKSDNAIIKILHDFSTNVIEERKKIGSDSKKISSVSYSGKKRLA
MLDLMLAAKNEGADIDDEGIREEVDTFMFEGHDTTSMAICYSLMALANEPEIQVSFKFIREYF
KHLLSNKSSSRIDLFFMFQSHYPT
>PverCYP347A4
MLLILLAIILVLAYYLLYRNLDYWKKRGVPGPTPYPIVGNLGDIITGKRGAEEIYTDIYRKYEGY
PFVGVFKSIIPVLLVRDPDLIKSITLKDFTSFQNNDIFADKRIDPIFARNPFALRGNEWKTKRAQLT
NCFSSGKIKAMYPVVDNNSKNMVRYIDNEMKNTNCFDAREIFVRLMLDNVAACALGIEGKCF
DEPYSDIRRMADNFFFTEGLSSLRFILCFNLPPLANFLKVKVITQEVEDTLMSIVSSTLKYRKEN
NVVAHDFLQAASQLDSSNSIFNEVDITAHVASFFTDGYETSSRTTALLIFELAQNKDVQDKLRAE
IVEAYKKNGNQFDYESIMKLEYLDACVHENLRKHSVVPLMPKICTEKFTYTPTNPEYKKISVTI
EPGTSIVIPSTAIHHDPKYFDEPEKFKPERFLNKDVDKYVYLGFGLGPRMCIGSRFAIAQMKVGL
AYFLKNYEFTVSSKTETPLKYDPWFFLLSVKSGLWIELRKIEDN

>PverCYP345A1
MILTSSWILDFLVLSVTSAVLLYKYSTRKFNYWKKRGVYYPKPIPFLGNTADVSLFRACIGEWL
KRIYDSTDEPYFGIFVFDKPILVLKSPELVKQILVKDFHNFSDRSVAQPKHNKIISNFMFFQRNPE
WKEDRSRLSPVFTSGKLKMMFNLIQGATENLSNYLEKNEGEMESKEVFSKYATDVIASCFFGID
SHCFGEGESTFRKLGRTIFDFSWRNGFVQTAYFFRQNWVDFFKLDFIEKSVLDYFHGVFSDVLQ
ARRTSGIRRNDMIDIMNEVNNNEKHRSNTDAEDELRLVTQAIQFFAAGFETTSSTITFTLYELCL
KKGIQDTVRKEILASIEKHGSITYEGLMEMKYLDMCVKETLRKYPVLPFLDRTCNEDYRLPGT
DLVIEKGMTVFISMFGSHFDEKYFPEPDKYDPLRFQNNSFNSEGLVYIPFGEGRRSCIGERFGLM
STKASLAYVLAKFEVEKCARTPDPMIFDPKSVILQSTVGVPMNFKLIRE
>PverCYP348A5
MSMYFISIKFSYVMLPYFLILDITSLRNLFCIFLFKQSSYKVNAIVELVVSNPAKKNCNAGPITFS
VSNFVSEEYFSSFSEFVQYINQIVLSGTVCLSSSYCSRNRHVKPIQHPFLNEIHLEEIHPILPEEVR
RLYETIPPREIEDIFAEFPKHCIFVVEAVSIYTEETFRYDVSGVFGKYFLRFQLAFVFFQVVEEVY
TDVLDQRKHHLKIFTQYNSLHEHNFQKDKCSVYQVK
>PverCYP302A1
MGSGVFSHIFFYFEFCQGTYARGRLRAHHSKSFHLYNFLGPSPKGYVNQAFLVEILVLESLWIVF
VWSPGSIFSSRCSPEHGNIKQEKPFSITRSVPGNR
>PverCYP349A1
MKEKEFSNNSCFEEGETRRKAFLDYLIEIRNDEKKGLTEEELEDEVNTFIIAGSDTTASTISFTLI
MLGLHQDLQKKVHDEIIETIGYDRPVEPSDLPKLKYTERFIKETLRLFPIAGIIVRAVDEDVDLG
DHVIPSGCSVVFGILKIQTDEKYWPNPFKFDPDRFLPEETTKRHPCTYVPFSYGPRNCIGPRYAM
MVMKTILSHVLRRYSINTSYRNVEDISLKANLVLRPKDGYNVSFKLREG
>PverCYP349A3
MKSAYLENLQPQNIQHFLLLVCSATILVWYLNFLWKRRKLYSCANKVKGPFSLPFIGNALMFIG
DGNDIIKKLLKIQQDFPGLSKIWLGPKLIYLVSQPEHIEIIMNSPKALEKDDLYRFTKDAIGCGLL
TAHAKKWRYHRKLIAPSFNQRILDSFIEIFSEQSVIFTEQLTKYVGRRNFDLYALVTNCTLDIICQ
TAMGVNMSIQKGDSDFGEVLEKMMELITYRVFRIWYHIDLIWKLSKYSTVHDELVKKFHETTS
AVVRKKLLEHNRKQPSCVYAEIDDEPTKKRMAFLDLILENSDFTEDELNDEVTIFLAAGTETTA
SSFCCLSTMLGMHQDIQQKVYEEVIEVVGPDRDVQPDDLNKLKYTERVIKETLRLFPIAAFFAR
FVEKDIEIGDGITLPAGSTASFGTVHIHRNPDIWLDPLKFNPDRFLPEEVAKRHPCSYIPFSYGPR
NCIGWRYALANLKTIVSTVIRIYKFHTEYKSVEEIELKINLLLRMRD
>PverCYP348A4
MMINIFNSQDAVSEFFRTFIKCQGHLLYFNDIYGHLIHETLTSNSLSNSTHKMPCREIEMKLSQK
ETWKPQSRLKIINCYF
>PverCYP349A6
MENQTTENEAIHYRIFLGLISSILIILVYQYIRFIWGRRKLYISSWNVPGPLSFPLLGNIHLFYRGS
DVFVKLTENLARTYPYIFRIWMGPHLLYCILKPEYIEKILCNKLEKSRWVYDILSTDFTGDGLIFS
RVTTWKRHRKLIQPSFGQKTLDSYIDKFNERADILRDLLQKHIGETSFDLRKKFFNYTVDIICET
LMGVNVDFQTTNSGAAKDLSRILQILVTRIYCAWYHIDLIWKLTPLYKELCHLNKKIKKEYFMC
AIFERMNEPKNSIFMDDIQDEFQKKLPFLDWILQTTDFTETEMIDEVYTFMGAGSDTMTSALNF
CVIMMGMYQDIQERVYEEVLAVCGPERRISASDINQLKYFERVVKETLRIFPPAPFILREIDADL
DLGELVLPKGSSSMFIIRTIHTNPKYWPNPYQFDPDRFLPENVARRHPCAYIPFSYGSRNCIGFRF
AMMEILTTLSILIRKFKFFTEYKTIEEIELIPSIVLELKYGEKVRLELR
>PverCYP347A2

MILFYLPTVGTFLKLKVISDQMEDKLIGMIKATLEYRRENNIVRNDFLEYVSQLKNGTEMFTDV
DIAAHAASFFGDGYETSSRVMSFLLFELANNPRAQDTLREEIMESYRKNNNSFTYESIQEMTYL
DACFNESARKNSVIHQLTKICTEKYTYTPTNPEYKKITVTVEPGTPIIIPIMGLQNDPKYFESPDE
FRPERFFDKDNIQKYTYLPFGEGPRVCLGQRFGVTQIKVGVVHLIKNYKLTVNQKTQLPLKYD
PWFFLLNAIGGLWVDLHKINN

>PverCYP348A3

MTGSTCSMNRFPGQILLPVPKGVTRKVALLDVSDFSLSSHLSGINSLALSKYSLFLQAMPVWH
KTTLPALILYPDKSKSVSVILAKQEAVGRILNDSNIQALKYDFDESSRIFCCVPSFFAIRMLEIIIGV
AVVVALFYYFAIKPMSYWKNQGVRQTNAKWLVGDNGGTLFKTESFVEMIKRIYNDCPEATRY
YGMYQFAIPTLMIKDPELLKQITVKDFEHF

>PverCYP4G14

MSTTTASPDLVAPSTLLSASSVFYFLLIPAAILWYAYWKISRSHLIELADKIPGPKGLPILGSALQF
LGSSPQIFKRVYELSFQYGNVVKVWAGSKLMIFLIDPRDVEIILSSHVHIDKASEYRFFKPWLGD
GLLISTGQKWRAHRKLIAPTFHLNVLKSFIDLFNANSREVVQKLKKELGKEFDCHDYMSEATV
EILLETAMGDQSGYDYAKAVMDMCDILHLRHTKVWLRPDLIFNFTRYAKTQEGLINIIHSLTRK
VIKKKRSDFEKGIRGSTAEVPEELKTQNTGTVSSKTVVEGLSYGQSAGLTDDLDVDDDIGEKK
RMAFLDLMIEASQNGVVINDEEIKEQVDTIMFEGHDTTAAGSSFFLSMMGLHQDIQDKVMQEI
DEIFGDSDRPATFADTLEMKYLERCMMETLRMYPPVPIIARQLRQDVKLASGDYTLPAGATIVI
GTFKIHRLPEIYPPDNFLPERTANRHYYSFIPFSAGPRSCVGRKYAMLKLKILLSTILRNYRIISDI
QEKDYQLQADIILKRAEGFKIRLEPRKRLAKAI

>PverCYP9Z1

MDHRTFIPEDADPLWGKNLFALTGQKWKNMRPILSPSFTSSKMRSMFVLMSECSETFTKHFLE
KKEDVIELEIKDMFTRFTNDVIATTAFGVKVDSLAQPNNEFYLMGKSATDFTTIWKNLKFFGY
MFVPSLFKFLKIGLFDASVSKFFVDLINDTVKTREEKNIVRPDVIHLLMEARKGIQQKEENNLPE
AGFATVQEVDLGKGGSVKEITNLDITAQALIFFFAGFDTVATLMCFLCYELAVHQDIQTRLREEV
QETLENCNGNLTYEALLKMKYLDMVVCETLRKWPGAVAVDRICTKPYTIEPSLPEEKPLHIERG
TVIWLPIIAIHNDPNIYPDPDKFDPERFNDANKDKIDPYTYLPFGLGPRNCIGSRFALLETKTVMF
HLLKHFEIVPTGKSRIPVKLSKKSFNLLAEGGLWLGLKPVKV

>PverCYP6BJ2

MYSLTAPIFMPLDLDLIKNIMSKDFNHFVDRGIYVNEKVEPIGAHLFAIGGLKWRNLRTKFSPTF
TSGKMKVMFRAIANCGKILEDYISREVVKQEPMEIKSVLACYSTDIIGSCAFGLECNSFKDPDT
PFRKYGDRVFRQTRWEALKLSFSLQFPEMARVLRLRIIPKEAEDFYTKVVKDSIEFRERENVVR
KDFLQMCIDLKNDEGSKGDGTSLTLNEIVAQCFVFFAAGFETSSTTMTFALYQLAVNPDIQDRV
REEIHSVLAKHDNRLTYDALGDLKYMGQVIDETLRMYPPVVFVTRKCVEEYKVPEHDVIIEKG
RRVFIPIRGIHYDEDYYEEPQVFNPERFSEENKQGRHSYAHIPFGEGPRICIGMRFGIMQTKVGIL
SILKNFRVKLNKKTVTPLKLSPTSFVPTTVGGIWLDLEKLK

>PverCYP334B1

MFQKMEHDGFRLQKGKPRTDTIPGAQSEREVGVWIDFVLVGIVESLWIELVWVWVDVRVVVY
GNT

COE:

>PverCOE1

MGAQVFLILVFYLIEGMEQIIISSSEAPSLIIPDGKIKGTLETTLDGRKFCAFQGIPFAKPPVG
DLRFEEAVPIEPWEGIFEANTAYACAQMDYNSPVKARRMKGSEDCLQINVYVPGGNINES
RNYEVLLQIHGGALMLGDSLSLNNPLYIMDELDDMIFVSFNYRVGPLGFLSTGDEVMPGN

NGLKDQLLAMKWVKKNIGYFGGNPDSITLSGHSSGAACVHLHTLSPLSRGLFHRAHIGSG
SAFNPATIKEKPLVFARKLAAILNCSSESSQSLKECLKQRPVSSITTVTDQFFGYKSLPFAPFA
VVLDKESKNPFLPKLPYELAKAGGMLDIPLIFSIVRDEGIYPAGSYYEDSENIDSIWEEVAH
YLLDYNYTLPVKDRVEISKKIKTLYLGPGTRISSENFFDFVKIFSDRYFVIGAEAAAKMHSV
ASKSPVYFYYFNYQSHNFSTSTLYCSKDIGGIAHSADERYITGFKMTRSLFKNDQEIKERLY
QMYSSFAKTGVPSLVGLDSWLPTEGNILTYLNMTSSSDIKMEKTTSFSPYAEFWRSHGLAE
NERDVFSDENYPSRSGSANDNSHDHFWISFLVFSTKAFFRFRGYF
>PverCOE4
MGRCSLGQRKSAKHSMEPLAQSHGGLYFEESLVSDIADALAWPQVQILDFLCHQRAILLW
QGVVIVEKKVCHFQKHRIHSLELVQEIESWVQPFIPHSRNQQRHISDVTFPYYVIRQLGQE
GVAGVFLDHRRERRERKMAVAIKLPVVVDKVAGRTPFEARLEQPRILSRAAQEGSHFPSM
LLKNIKKYSPGAIGGLGNVILSSVKHRTRNETPHFTAGFSLMTHGLKATPLPANALWNKPL
DMGERVCKWTLAPPAEWPIRVTESALPPKHPILFFTHFIARTWSFIALLPGMTPSSVLRKPG
TRVQYRESVSAV
>PverCOE5
MFIAKARFIAKANSTSYTSMWFVSSALFIFFALLIAISSEVPEEIPLINTPLGIVRGTWQKSFE
GRRYSAFEGIRYAQPPIGNLRFEEPRPIDPWSGTWEANRLYRCPQNVAHGSGDEDCLYINV
YVPETSKKEKLEVVAHIHGGGFEFGSANDFLGPKYVMDRDIILVSFNYRLGILGFLSTQDE
VVPGNNGLKDQLLALKWVQENIGSFGGDPKLVTLTGFSAGAASVHFHYFSPQSEGLFQRG
FSLSGSAINLWAIQKRPESKAKTVAEAVGCPVTNTRTMVECLKSRPAQLLIDSMESLRPITH
MPACLFTPVVEKNSSNPFLVEHPEEMLKNGKVLDVPWMVSVTNNDGLVYLLWNEDNLE
KMHSQWEEVSSVVMDYDGIIPRSQIKNYARKIKDFYFPTGHPTTKENFHNLEKMFTDWVF
LLPIEKAVTMQSKVTKSPVYYFRMSYVGQNSLKYLLPSRYAKDVEGTCHGDDMTYFFGGI
VRNDLSEDEILLKNSCLDMLYSYANKGYPVFYDTELKKIGDGELFFYNISGPQDIELKYITE
TAAKSLWTDIVNSSIYDAKDEL
>PverCOE7
MRVFAQMISLWISVCMAFHSIGGEVTVEEPIDSNTIQRFTQSIEQNKTKENDVKALKKVFL
SNRSITCNDGSQAGFYLRKSHSSKKWIIFLEGGWYCYDNHTCRNRWLKQRHFMSSLKWP
ETRDVGGILSPDSEENPFWYNANHVFVPYCTSDTWSGTKPPSSAQMFSFMGSFIVRQVVR
DLVPLGLENSTDLFLAGSSAGGTGVMLNLDFVKELLHEEMTLTHINVRGVSDSGWFLDRT
PYMPTNKPAVEAIRKGMELWGGKVPRRCREEYMNEAWRCYFGYRLYPTLTTELFVFQWL
FDEAQIDVDNVGAPVTKQQWDYIHKMGDGLRQSFENVSAVFAPSCISHAVLTKRDWQKV
EIGDTSIAEALHCWEQKTSRRRLKRLKRLKIDAQYNESPKVAQKKKKKRNDGAGLPNTPP
DVDGKAKRRRRMKKNRRGRKNKANEHQLSLNENRASPHYTKKNSSQNRPIRSVNQGPK
RPGKRHCSHRRLERCSWPQCNRSCPKLHNPFTGEEMDFIELLKSFGLDMNSVADALGIDM
PTLNNMDHSELLNLLTQQSNY
>PverCOE2
MMRVVLFLCFLNFAYSRTGPEVETPLGKVQGLYKTSYNGKPYSLFEGIPYAKPPIGNLRFE
EAVPVEPWIGVWNATRIPQRCMQTEISGIGDVRGSEDCLYLSVYVPGDKIDPTKKYDVLLQ
IHGGAYMIGSGTDVAESSFVLDMDLIYVAINYRVGVFGFLSTEDGVIPGNNAMKDQVLAM
KWVKNNIGCFGGNADSVTLIGHSAGGASVHLHTLSPMSRGLFHRAFAGSGVALSPWVIKE
KPAEHARKMAALLGCPTEDSRLLKACLKGRPAGDFVDNYREFYGYGHLPFSPFSPVVEK
NASNPFLTKLPYDVIREGDVADVPLLISTVRDEGLYPAFYFLDQFEAVDPVFLEVAHLLFDY
NHTLPEQNRSEVAQKIKDLYLGPGQSISNATYKRFFKIFSDRDYIAPSEMIAKAQAKVMQS

PVYYFYFDYNSRILPTSILMAGRNLDVVSHTGDTIYSLGQAMAGRRFTGDDDKMKDKLQ
NIITSFVRTGYPSFDGSNTWKNVGKDSFLTYLHVVGPDYMRIRKVKSLGNSDFWRQFGLG
EFEREVTV
>PverCOE3
MISKVPITVEIMINEVFQENLPRAHNLITNASSGIRIENMFQPFTDSLYVIGTYAAIRNHVRS
TNLPIYLYRMSFVGRLNTLKVFANMTHLPGVCHADELGYLFKNSALPDLPTGSQEDKTIR
MFVRLWTNFATFGNPTPNSRTQEQLNVTWEPVKEERNCLLDIGDEPVLVTNSASEESRIGF
WRDVYQHSPKTKHFL
>PverCOE6
MGLKDQLLALKWVKENIQYFGGDPDKVTIKGQSAGAASVTYHILSPSSAGLFRAAIASSG
TALCNWASQRPDGRIKAYRLAKEIDPSINRYNTTRDILNLLLTADADRISQTGTTFLPVVEV
EHEGAFISEPMYDTVKNGRINKVPLLIGFNSEEELAKAKDIDGLKRSAESYDTHPEYLVNA
DMNISDPQTILEVAMKAKPMYTDSTFIDDLGAVVKFTSDDQFVRGILRYAQLQAQFTDVF
VYEFTYHGILGMNNFTFDGCGRVMHGEEGKYLWAGTDLTKVPESDVMTLDRFVGYHTN
FIKSLDPNGEDPIFSNLTWPKVNGTNYWYMEIDKDLTVKENPRDSSYRGWVSLYDAYAVE
PLVEI
>PverCOE8
MTGETEDCLYLNVYTPVKPGTNASLPVFLFIHGGGFVFGSGTFSEFGPKYLIDGDIVVVTL
NYRLGPLGFLSTGDTGISGNMGLKDQHLAIQWTYDNIQLFGGNPQDIVVGGESVGSFSVG
QHLLSPKTEGLVSGFIQQSGSHLSVESMNLHPRKYAFELGRNLNSTLSDEDTVSLIYTLRN
ASAEEIVEAANMIYSPDEPSGFMYGLIWSVVIEDSNSSEPFLTSLVYDDMLKGKFNKVPSFI
GFNSEESLLFMPANETLLEIYAAQFDTDPSLLVHPSINVDNRTLVGEKLKDLYTNSSFAVDID
AFIRFTSDTVFVRSSIRQAEITSESVPVYLYELSAGENINDEFPGFAHTLDLLYFWDSSSTYT
VDEEIREIALKLWWNFIKYKNPTPEIDPALQNIEWPTFDAENINYLNLNHTLSIETNPRNYR
QLRDILLEYMKPPFFAF
>PverCOE9
MSAPVVESEEGKIRGCVGSDIDGNPILSFLGIPFGKPAIGHLRFKAPEPVESWDGVLDATKV
GPRCCQMNIFSGEILGSEDCLNLNVFTRKLPTKDDPLKPVMVWIHGGAFLHGSNETEMFG
PEFLMTADIVLVTVNYRLGFLGFLSLQDNSLKVPGNAGLKDQNLALRWVQRNIKNFNGD
PNNVTLFGESAGAASVHYQLLSPANEGLFHKVILQSGSALNPWAETRQSVVDFVKFMGK
DVNNEKAALQYLQNIPAETLVVEQQKFIASQETGLGIIGPVVENSNESELISERPLNLLKSG
SYRQIPMIIGYCSHEGLIAKTDKIFHVPHLENENHNLDLFIPWIMNIEKDSDESKLVCEKLA
NIYFKEENIQSKYLLPSDFYFISGIVASALQHANTSNNPVYLYRMDLDTDINFLKVVSDLRE
YSGCCHADDLGYVFNITSVPGMKLGEVETKAMRRCVELWTNFARYGNPTPPENKTLNIE
WKPIEPNHLHYLSIENDGLFIQENPDSERMDTWRDIYNVSQFTSGLL
>PverCOE10
MVLGLISGVIQQSGSHISAGGLAFRPKDNAMDLGRLIDPSLSNNTSHLARTLRQASVEDLV
KAADELSHPPPMGFMFSMIWIPVIEDENGEEPFVTSPMHEDTMNGNFNKVPSFIGFNSEES
LWFVSSDDAAMQRSAAVLDEDPSLLISPGSNVENQTLVGAKLKKAYTDLSFQEDIEAMIK
FTSDIYFIRAVIRQAELTSKYIPVYLYQLSVSSYENDKYTGVPHVMDLPYIWHLPAFPEVDE
EVREHMLKLWWNFIKYKNPTPEKDPALQNIEWPTVEPDLIKYLNLNKSMSIMEGNPRNY
GMLRDILVEYMKPPFRTF
>PverCOE11
MHWCLLSFQIFLIIFSVCQGRPQVRDYIEVAIPDGRILGRLLKSDTEVPYHAFQDIPYGKAPI

GDLRFKEPEPVDRWDGVLDTTANRKICVQFDSVTNDSRETEDCLVLNVYTPSLPNGRDPH
QPLPVLAWVHGGSFSRWSGEFASFGPDYFIDQNLVVVTLNYRLGPLGFLSTGDNVISGNY
GLKDQLLALQWINKNIEIFGGDPNKVTLMGQSAGAAAVGYHLTSEKSKGLYRAAIMHSST
HLTCWALQKYPKYAAYQLGNSLFNDFNKNMTSEDFLKLMRDVPFKDLKAHSAISLPTSM
ANCQIPTSIAWVPVKEDPNNKNSLVVGMNHENLKNGNINKVPLLTGINSQEVLLFIPSLIA
MVYLGSAIDLNPSLMITGNMNMKPANRNLAAAELKPIYTDLGFGLDTAALIKYASDALFN
TPLGRHAHLQSKYTDVYFYEFSHKGPLGQLNTTYPGAEGVGHEEELRYLFRDANNADLS
EYPEDDVVTHQRLVYMWSQFVKNTNPTAEKNELIQNVDWPKVTQDRFSYLNINSSLSVS
VDPKNYKQWSIIIEEYAEKLLDTY
>PverCOE12
MKVYITKPVDNQNRRVMIRTILIALGVLILIGIVIVVLLIMQQCQCVNNPITNVACNDSSQQ
DSCTLVSIPNGKLRGRKAFSPRKVPFYAFQQIPYAKPPIGELRFKPPKPAMNWEGILDATKN
TKTCYQFKNVFNITNWAETQTEDCLFLNVYTPVCPTSGSSLAVMFYIYGGGYLSGSAEYDI
IGPDLFMENNIIIVTLNHRLGPLGFLSTGDEVLPGNYGMKDQVMALKWVRENIKYFGGDP
DKVTIYGQSSGGSAVGLHTMSKLSKGLFRGAICQSASSLSTWSYQGNYKEIAYGIAQNLN
SSFSLNATSEELLKFLLSYPAAEVNAATRKFPKKFLDEMYYQGFVFTPVIESPNNENAFITE
SMYSAVKEGHFSKVPLLIGIVSEEAINEFEVPLPEATEKWKERAKEMDEDPARLVTENMHI
NDESIKRRIGEEIHEMYTDGLFSNNLGKAVQYLSDATFARAVIRHAQLQSNFGDVFFYQFS
HYAPILGERLNFKDSYRVGHSDDNPFLWVFNKRNIFKESKEPMDILTSDRYRALFANFITHL
NPTPNRTELFDNIIWPTVKPDNFLYLNINHTLSIERNPRNNTYAKWVDIFEKLAVKPLNTY
>PverCOE13
MTMNSRWRVVLILLKLCGDIEKNRVEGLQRFVLSEEDVILSIRDGLVMGRKARTIGYNRS
VYAFQGIPFAEPPVGKLRFEAPVPKTPWSGIMNSQQDNDVCVQGINPVVGSEDCLHVSVY
TPSLKDNLPVMVYIYGGAFIIGDSRYNTTGPDYILEENVIFVQFNYRLGIFGFLSTEDLVCP
GNNGLKDQLLALKWVQQNIRFFGGNPGKVTIFGESSGAAAVGYHVLSPRSRGYYRAAIM
NSGSPLSLWSLNRNAKEIAFLAGSTLSIATTDSKVLIERMRRFNYTYLQQVASTISTIVTEM
NPLEGVYFGPVKEPNHPKAFFTGESDEMFSTGKFNRVPVLIGVNSNEVAAVGFITELWRLY
ITERHPKISDLTPADMTDDANKKFYAGLDIRCFYFDCFSLSLQPDDKIITVR
>PverCOE14
MMSEDLVVSVEQGRIRGKIQEDYHGGSFYSFSGIPYAKAPVGELRFKAPQPAEPWEGVRN
GTKEGFECPSLDMYLKYHIGNEDNCLNLNIYTKELPRETIFRKPVMVFIHGGGFLYGSNKQ
AFIGPHYLMTDDIVLVTINYRLGVFGFLSLEDQSLGVPGNAGLKDQVLALKWVQKNIHNF
GGDAKNVTIFGESAGGASVHYLTLSPMSKGLFQKAIMQSGCALNGWARGTSNFSYISKM
MGYKDTDEKTVFQKLCRASSRSLINAQFKVEDSFFPSAIRPFGPVIEYPHEGAFLAEDPEVI
LKSGRSHQIPMIMGYNSMEGIFYEAIRRSRSDANLPKTLERDIPYNMNIPSQSEKAKQVSR
MMKEFYYRDEDILEENIENRYLLVSDTHFVHGIQKSALLQKLHSTAPVYLYRMSLDSSLNF
FKKFCEVKYFKTMIFVTMLTKLSGSSSFKQIFQNLNDKLPVKKMNGVAHADDLFYLFSTF
FSPAITKGSEEDKNIQKFVKIWTNFARTGNPTPQLDDSLDKCVWKPLDGEVIDNIFDINERN
TLTENLEGERMKFWDRLYSEYAVKV
>PverCOE15
MAYDLATFVDGKFPRNASSEQLLEFLRLVPANDLNVFAASYPQELLDNQIINGFVFAPTVE
PEHESAFITENMYEAVEKGHINPVPLIIGICSEEAIDRAADLEGFKWVANEMDLNASMLVN
PNMHLRDPERIQAAGEAIHRIYTEGDFIDNLGKAIRFFSDTSFNRGIIRYAELQSRFSDIYFY
QFSYYGAIVGERPNVKGAGRVGHSDDNMYLWTFGNWSGMNYQTTADILTFNRYTTLFTN

FAKFLNPTPEPSSLFDNIIWPTVDPDNFQYLDINDTLSIKKDPKGDIYPNWVEVYERMAIKP
YTTF
>PverCOE16
MYDRDHRRDWTSSKKFSVDMSETTLVEVSEGVLRGCQGANLDGEAFFSFLGIPYAKAPV
GDLRFKAPVSAETWKGVRDATKEGNRCYQLDVFRGGYQGSEDCLNLNIFTKKLPTANHT
DDLTPVMVWLHGGGFESGSNSPSQYGPEFLMLEDIVLVTINYRVGLLGFLSLQDTSLGVP
GNAGLKDQCMALKWIRKNIARFGGDPGNVTIFGESAGAVSASFHVVSESSRGLFHRAILM
SGCVLNPWACMEARRAVDLFREVGRDVGGEEEALGILRNMSVHEVLDFQLKYGEAIQAI
EVKFGPVIEQPNVTAFIQKNPMELLNSGNFNKVPIIMGYCSEEGLLFEFYKAMKGEDKRK
KIQRNELNLDEYVPKELKVDAGSEIHELVKNKLLKEYLSDESKFDVHAVSYLRKCQKNSS
TADMISKNFEQKVEVVPIQGVP
>PverCOE17
MKRFDASIKMTKNVFQCIFSTNGVKVMTMILVGCLAILWIYIRNTYANIVSIPNGEIMGGT
GRTVDGNNIYYYFKGIPYAEPPIGDFRFEPPVPKRSWNEIIDGTVEGNICIQGNDPVIGSEDC
LNLNVYTPSLTNRSKPVLVFIHGGGFIYGNTTYQSYGPDYFLDEGFVFVSMNYRLSIFGFV
STGDLASPGNNGLKDQQLALMWVQKNIAHFGGDPRRVTLMGQSAGGASVAYHLQSHRS
TGLFQRAIMQSGTTLNLFSLNKRAKETTFNIGDALSISTKNSTELIHELKKIDPTILQSKAFE
ESNQVFSRNTLAGIPFGPVMEPIHDGAFFTTGSDDLLRKGNFQRVPCLVGVTSNEAASQFV
IDVFGDLKMENAQLAPRDLTEDAIRRIEAGNAIDCFYFDCSLNTSSLENKVKFLSDDQFNR
PIRRFVLDLSRFISVYFYVLSYEGTLGGIENRTLPGVQHFEDLGYLFRTFFKEIPQTDVLMR
KRMVKMWSNFAKYGLV
>PverCOE18
MFSNCDILRKPKIYFTILGLILGIVLIAVIVCCYQTKINIATSKNDPAIENSCNLVKITNGQLK
GRTMISSKGQEFHAFLEIPFAKPPIGELRFKSPVPPDNWNGVLDATNNTKTCFQFLEYFHVA
NSEKSQTEDCLYLNVYTPVCPSAGKSLPVMFYIYGSGFLSGAANYDVYKPNHLMDNDIV
VVTSSYRLGPFGFLSTGDEVIPGNFGMKDHVMALKWVQKNIKYFGGDPKKVSIVGHSSG
AASVSFLLLSKLSRGLYTSAVSLSGSFLNAWTYQKYHKRIAFQLAAYLDDTFSMNNTSEEL
LEFLRGVPARQLIDVLGKFPDQFLDGLSVQGFVFTPVIETEHEDAFITESMYTSLKEGNIDR
VPLLIGMASEEALCLVKGSIPEQMRKWKERAARIDADHAFLVLDNMNIIDDDEKKKVGEA
ISDLYPKGSFVNDLRKTIRYFSETNFNRASIRYAELQSNFNDVYFYRFSYFSPSYPKHPSILG
TGGVGHGEENIFLWIFDGHDIYERADPMDLLTSERYRTLITNFIKYSNPTPHKASLLNNIIW
PKVQPNNFQFLDINETLTIQTNPMNQTYRAWVEIYEKMARKPLMTF
>PverCOE19
MHDNWDGVLDCTKNTRICYQFHENPVTYGDEPRTVPNLRESVDVQESEDCLYLNVYTPV
KPGSNNSLSVFLYIHGGGFIYGDGTFYTVGPKYLIDGDIIVVTMNYRLGPLGFLSTGDSVIS
GNMGLKDQHLALQWTHDNIHLFGGNPKDIVLGGESAGSYSVGHHLYNPKTEGLVSGFVQ
QSGTALSAGSVSLEPSKYAFELGRVIDPTITRNTSHLSYALKKASPEEIVKASGVITCPISIGFI
AGITWTPVIEVADASQPFLTSPVYEGMMKGNFNRLPSFIGFNSEESVVFLINDESLLQETAA
KYDEDPSLLINPVMNVVNRTLVGEKLKEVYTSYSFEKDPDALIRFTSDAIFTRPTIRQTELT
SEYMPVYLYELSVGMDLNRRHPGVGHSLDLYYLWDNIKFPREQYPVDLEIAETVLTLWW
NFIKYKNPTPNKEPILQNIEWPTVEPDNIRYLDLNKTLSIKTNPRNYNKLKKVLLDYMIPPF
YVF
>PverCOE20
MQYDHFTPPEQDMVSGDEDCLYVNVYTPSLNRSKLLPVLVYIHGGAFMFNYGGLYGPQI

LLDRDIVYVNLNYRLGPLGFLSTEDDIIPGNNGLKDQILALKWIKDNIEYFGGDPNSITISG
MSAGGASVQLHYLLPKSKGLFARGISQSGVALNPWVLVEDPLDTTKSLSASLGCPIEDNES
MVNCLKSRPGRQIVHAVKQFQPWLYNPFSPFGVVVDKWASNPLLPEHPYELLKDGKVLD
LPWLLSYTSAEGLYPA
>PverCOE21
MIWIPVMENKMADGAMVTSPMRQSFMDGNFNLVPVLTGITSEESVHYLSAIPSSTFNDSA
RLIDNDPSLLVHPRLNIKGNERETVGSLLKATYTSSTFTEDQLALIRVRASTFTRFKRHS
>PverCOE22
MSVKYSILISVCVFVKIVTTTVVNDLIVELAEPKGRIRGHILQSAKGKEFYAFQEIPYGKPP
VGEKRFAPPELHDGWNGTLDCTENRKICYQFAFPMTPINKDWEQSLKNVEANGELEESED
CLYLNVYTPAKPGTNNSLSVYIFVHGGAFAIGDGTLRSLGPKYLIDGDIIVVTMNYRLGPL
GFLSTGDHVVPGNMGLKDQHLALVWTRKNIHLFGGNPQDIVLGGESAGAYAAGYHLLSR
KTKGLVSGIIQQSGSHIGVAAFSLNPSKHALELGKALMTSWFPENTSSLVSILKTASPEQIVK
ASDSVQHPESWGFINSIIWEPVIENCDDCQALISSPMHEDMMQGNFNHIPCFIGFNSEESLG
FLSPDASALEWAAAQYDDNPSLLINSAINVENRTLAGEELKKTYTNSSFRENLGAFIRFTS
DVNFIRAAIRQAELVSKYVPVYLYQLSAARNIRDEYPGVTHLLDMRYLWDIPPLAADTSP
VDEDVREIVLKLWWNFIKYK
>PverCOE23
MNYRLGPFGFLSTGDEAISGNMGFKDQHLAIQWTYNNIHLFGGNPEDIVLGGESAGGFSV
GYHLLNPKSAGLISAYMQQSGSPLSVEGISLEPRKTALELGRVIDPTISDNNQELVNILKKA
SPEEIIDAAFRITEPSSAGFIHGLTWIAVIEDENNPQAFLTSPMYEQLLKGNFDRVPCFIGFNS
EESLYFFQSVEPLWQTYAALYDNDPSLVIMPSINVENRTLAGEKLKKVYTSLTFQEDLGAF
LRFTSDAVFIRGIIRQAELTSAHVPVYLHQFSAGANNSEEHPGVPHAADLVYLWDFNNISV
NEGIREILLKLYWNFIIHKNPTPEKDPVLQNIEWPTVGQENVIYLNLNDTLSIETNPRNYNN
IKKILEDYMIPPFYVF
>PverCOE24
MVFIHGGGFTSGSNRSIICGPQYLMAEDIVLVVINYRLGILGFLSSEDPALDVPGNAGLKDQ
TLALKWVRRNIEHFNGDPGNVTVFGISAGGASAHYQVMSPTSKGLFHKAICQSGTVLNP
WPYGQRNLLEFIKLVNQDCKTEKTALEILRNLPIEELYGFQDKFLDDKPPLGIFGPVIEKPN
PTAFITKHPIEIISAREYNKVPIMFSYCSNEGMLSEVYKMIASATGKTPLVMGLEDYIYREM
NVDQDAEKKAGICQLLSDFYNREDNSKDRHMLSSDYYFVAGIIAAVLNHARTGEDPVYLY
RMSFNGDLNLMKQLFPLKYTGAAHGDDTSYIFSFSPPHIPRKEIGEEE
>PverCOE25
MIVVRIFLIGIVAALSNAEDDLVVQLWKKHGKIRGHTLKTFNGDDYYAFQEIPYAAVPIGSN
RFKEPKEAEDWEGVINTTENTKVCMQNNAIAITQVPAGMNMTEDCLYLNVYTPLKPDSG
DDLLPVLFWIHGGGFYYGSGAIQYYDPKYFMDYKIVVVTINYRLGPFGFLTTADDVIPGN
NGLKDQVLALEWVSKNIHLFRGDRKKITLMGESAGSTSVGFLQLSKKVSALAGGFILQSG
SPLSPYSYQDEPKHFALKMATALDSNFKSESSEDLFELLRNTSAENILNAYVPNDRYSINIIG
RIVVWLPIIEDEKYSNALITQPMYSSLLNGNFNHVPMMTGYNAEESFFFMRGKHQTPKY
>PverCOE26
MALKDQRMALIWTYENIQAFGGDPENIIVGGQSSGAFSAGYHLLNPRNKGLISGVIQQSG
SPLSGAAYMVSSQAYALSLGRSIDPSIASDDVEHLLDTLYKATADEIRATSRKVLNSYNLGL
SSSPSWGPVVESEDVEDAFITSPMHEAFSKGNFIRVPILMSFNSEESSIFLPELGEVYKEAAR
VDANLSLLVGESVRVENRTLVGTELRKIYTSKTFSEDPGAFIKYTSDVIFTNPMSRQAELAS

KYVPVYVYELSVGGYNNSLFGGVPHSYDLGYFWDMQEKKFESLTFDESVREYLLELWW
NFIKYKNPTPKREIEWLPVSPEEIRYLDIDYKVTMKANPRNYKAVKEIYEKYVKPPFYIF
>PverCOE27
MNVNVNLGKGLIQGCPVSSLLGHSHGDDKLKVKIEEGLVQGFMRSDYDGNPVVSFLGIP
YGKPTEGANRFKAPQPIEPWSGVFDATKPGPCCHQIDKTGFKGSENGLNVNVFTRELPKP
NTKPKAVMVWIYGGAFLEGSNEIEVFGPEFLLTADIVLVVLNYRLGFLGFLKLKDDSLGVH
GNAGLKDQALALKWVKRNIKYFNGDPDNVTIFGESAGAASVHYQLLSPTTEGLFHKAIL
QSGSALNPWAQTRHCAIEFVNFIGKNVKTEKEALEYLRKMPLEELLKYQGKYLQSHVTRI
GLIGPQIESPHGEEFISERPIDLLRSGKYQHVPVMIGFNSKEGLLAVANIGMFHTPYIEGDKH
NLDLFIPWFMDINKECPESKEICEKFANVYLQGDNAKDKFLLTSDFYFAAGIIASAVQHAK
SSKYPVYLYRMDLDTDVNFSKISDNLKEYQGMCHADDLGYIFNITCNPNMRIGEVEEKAV
RRCVELWTNFAKYGNPTPKGNKLGIDWKPVEEGHIHYLDITNEGFIVDKDPQIERINAWR
DIFNISPNTAGFL
>PverCOE28
MLGCKVLSIFCIIFTLVECGKVCEKDVVIQTTAGIVKGCFTSERTIGMKKPLYWFRSIPFAEP
PVGNLRFEAPQAKKSWKGVIDVSEPMPVCIQDSNPVRGSEDCLYLKVYTTMKPSTRNNLP
VMVWIYGGAFTSGSTDFSDHAPDHLLDQDVIVVSFHYRIGIMGFLSTGDGVAPGNSGLKD
QILALKWIKENIRSFGGDPDRITIFGQSAGAAAISYLLQTNQTQGLFQRAIIQSGSSLSPWGL
SKSTPDFIRSIARTLNVPTSSSEAILQGLKSIDVELLQTTSNSQMTRQLISSNPLSGLVYGPVL
EPDHPGAVITGRSHEKLANGQFHNVPIMVGYTSMETMIDSITAIMRVWLARYDLNPTLLVP
QDMNARATARAIIASSIKSRYFGLIPILTSFTNIMRFTSDDQFERPIQEAIRLYSAKTGVYFYR
FSYQGGLFGVTNRTIDGVGHTEDVGYIFHLNWNGTAEDLRT
>PverCOE29
MVEQNVIVVTINYRLGPFGFLSTGDEVMPGNMGLKDQQYALKWVQQNINLFGGDPNKVI
VMGESAGSASVTYQLISSSSAGLFRGAIGLSGAALNTWAYDADAVEKTYGIAAQIDPSFSR
DSTTQKLMDFLQSVDAKSIHATASNASYRSFAPVIEVEHEGAMITESMFQSAKEGRINRVP
YLAGICSEEAIILASDKHWKMIAGTYDKDMKTLVDSDFHITDETVLKQAAADIKAIYTTDT
WRGDIDAFTKYFGDNRYMRAPIKYAELHSQYADVYFYQFSYLGKMGVQDGLKGVGHGQ
DLRYLWAYYTNYDAFPEADMKTLDRYVGLITNFAKFLNPTPEASQLFNNVIWPKVTNGH
YYYLDIDQELVVKENPREFSYKKW
>PverCOE30
MQNEDCLYLNVYMPEYPVSNASLPVMFYIYGGGFVNGFAIFDTWGPHYFMEHNVIVVTA
NYRLGPFGFLTTGDLVIPGNYGLKDQQLVLKWIHENIKYFGGDASKVTLFGQSAGAASVN
FHYMSKKSEGLFRAGIAESGSIFSPWAYQENYKEIAYQLASNVDSSFPKTATSKELLDFLRN
VPAADLAKAAASFPVSQ
>PverCOE31
MNIVDEEVLLEVGSQIRQIYTNETYFVDDLGQALRMLSDSQYIRPVIKFAELLSQYADVFL
YQFTFQGKINNNHIDVEGVSGVAHGGDMKYLWTYYKNLDSFPLADVKTVDRYVGLSSNF
VKYLNPTPTPEELFGNITLPKVTKKVIHYVDIREELEVKTQPRRFSFEKWNKLYEKY
>PverCOE32
MTTPIVEISEGRLQGYSKKNLDNEEFFCFTGIPYAQPPVGKLRFKDPVPVGPWQGIKDARK
EGAACFQRDFFTNSYTGSEDCLFINVFTKSLPERNSLLKPVMVYIHGGGFIFESSSPRTFGP
EHLLTENIVFVTFNYRLGILGTTEMTFEKNSTCFS
>PverCOE33

MFYIYGGGFVSGAANFDFFGPHYFMEHDVIVVTANYRVGPFGFLTTGDLVIPGNYGLKDQ
QFALKWINQNIKYFGGDPAKVTIFGQSAGAASVNFHYMSKKSEGLFRAGIAQSASILVPW
AYQKDSREVAFQLGSYVDSEFKQDATSGELLTLLRSVSASDLNKAALSFPSDMYNNQIVQ
GFLFAPTVEPEHEAAFITEMMYEAVEKGHMNRAPMMIGFCSEEAISRATRISDMQWMAG
QMDLNASLLVNDNMHLTDPNQIQAAGEAIHRIYTDGTFTDNPGKAIRFYSDTSFDRGNIRY
AELQSRYSDVYFYQFSYYGPMLGNRGDLEGAYKTSHASDANFLWVYGNWSVIDNQTPA
DILTFNRYQTLITNFVKYLNPTPEASSLLDDVIWPKVQPGNFQYLDINDTLTVRTNPKGDV
YPQWVQIYEQMAIKPYTT
>PverCOE34
MNFLQSVDGKSIHATSTNHKYSSFAPVIEVEHEGAMITESMYKSVAEGRVNKVPYLAGICS
EEAILLAAGKNWKGTAADYDKNLHKLVDPDMYVTDETILEDASRDIKAIYTNRTFQEDIG
AFTQYYGDNRYMRAPIKYAHLYSQHADVYFYQFSYHG
>PverCOE35
MDGNIKKIPTLIGFNSEEKISVGKVKNKETLRQYDKNPSLLIPKYINMSSGNRSIAGNLLKK
VYTNTSFEEDIGAYVRWTSDTWLVTGVCKQVELGSTQSPHYLYQFSYKGDMGGNTPPIIIP
GCGTVEHQEELRYIWDNGSNSDLNKFPEEDQMTHFKFIKMWTNFIKYYNPTPENDRLLNI
TWPASNPDLLYLNIDTKMSISDNPRQFKQQKTVLDRFNEPPFIAY
>PverCOE36
MAERKVVLTALLAVIFGYWITTRRSYHIPGTKNVVQLQAGHIRGHTLLSKGGREYFAFQEI
PYATPPVGKLRYKEAIDPEPWVGVLDTTKNTKLCIQKMPIFDDQTPDPRENEDCLYLNVFT
PQLPVNKDSPLMPVVVYIYGGMFQSGDPTEYDFSLFLEADIVAVTMNYRGGALGFLTTGD
GVIPGNLGLKDQNLSLKWVQRNIHLFGGDPAKVTLVGHSSGAISVGCHLISKRSKGLFRG
VIMQSSSPLNHIAVQKDPRYRAYELGKSLSEDFTSQNTSEDLYKLLMSSSGEKICRTNIEIPP
HLNGVMLGVYPLLWGPVIEDSKDPNAFLNGPMHEDVVKGNVNKVPLLTGITYEEFTGIEI
KKQFQEAVDDNITSLAYHDANIKVENRLQYAIDLKHFYTDVPFAENKTALYKVIGDGLYTF
GIIEHAIQHSKYSDTYFYKFSYKGPINGVHNLINKEEGLNKTAHVDELPLLYSYRLNPHVTP
SAMDILMQKRIRKLWTNFVKYQ
>PverCOE37
MRKKQQLMECFTGLFQKAIMQSGCALNAWARGKPNVLDIGKRMGYTETDEKIIFDKLCL
ASAKSIVNGQPKLIDSISADEIRPYGPVIEYPNEDAFLTEDPEVIIKSGRSHQIPIIMGYNSLE
GIFYEIFRKNGSKLRKTLEKDIPFNMNIPQEKSEQVGQKIKEFYYRDEDISEDNIQHKYKLV
SDNQFIYGIQKSALLHKLHGSAPVYIYRMSIDTSLNMMKKLCLAKYFITMMFISFLSRLVR
NFSLGSILQNLSNKLPLKEIDGVAHGDDLGYLFTTYFSPIVTKGSEEDKYIQKFVKIWTNFA
RTGNPTPEVDEALDKCVWVPLQGDGVDKICDIDKTITIGENLEKDRMTFWDNLLKEYAEP
VRGL
>PverCOE38
MSDKKEIGEVKSEDKKDIEMEEREKMLNAENEAHNKETTQEKTEEKKVTEMKDGMEVK
PKKIPIGGIQMPGFFTRSKSKERCKEEDSVEVEVDLIEKSTESNDTSTTQTKVKLPNPFRKS
KTEEDGDKSTEQKERKKLLNAIRLPLVSVFPKKKKDDNLENQAQAGLASMETLDDKSTG
EKSTEEKNMKNVALDDKTDVENQGGDKDVPLTLKLKDYKVAIGLTILFIITVIIIAVMVFSG
KTTEKSRPLRDGKFMVTHTGCGQIEGTLEDGAYTFKGIPYARPPVGDLRFKYAQQLDSLQ
YCWNGTLPAHNATPPCLQILGNGTVVGREDCLTLDVVTPSVTYDNPVPVVVLIGADSLMG
GSPGRMMPSAGVAKAKEVVFVRPNFRLGALGFLALRALSDSDYPKTSGNYGLSDILAAL
KWVQLNIEHFGGDKNSVTVFGHKAGATLVAALSSTKDAKKYFSRAWASSGGASYPKKPV

AEAETENQSFMESVQCEDAACLRSLDAEKLVTAVEDTWRKQQADLPVGDEKPEGRHEW
LVLDGRILREYPEDVWSREEGLAVPLVLGTTAQSGATEKLLMKHTQWTEDLVKDHISRSL
LSSKNLTESTLKMYPANYSGLSSLISDIRIVCPLFSLSNRMPKVPFYVVTQPRAGLGYADAD
FDVDVILGNYKMARPEESVYVSAMQGLFYDFVFRGVVNQEMTGQKVLLVGNGVVPNST
YSHCNFWILKNVVPLFAALD
>PverCOE39
MGSVSLLVVCVLGSARGYSWPSEETTTRPSFYKDFHTDPLVVETTSGLVRGYSKTVLGRE
VHVFSGIPFAKPPIEQLRFKRPVPIDPWHGILDATKLPNSCYQERYEYFPGFEGEEMWNPNT
NVSEDCLYLNLWVPQRLRIRHHADKPPSERPKVPVLVWIYGGGYMSGTATLDIYNADIIAA
TSDVIVASMQYRVGSFGFLYLNRYFQRGSEETPGNMGLWDQILAIRWIKENVAAFGGDPEL
LTLFGESAGGGSVSLHLISPVTKGLAKRGILQSGTMNAPWSYMTAERAEQIGRVLVQDCG
CNVSLLEANPHQVMDCMRAVDAKTISLQQWNSYSGILGFPSTPTIDGVLLPKHPMDMLA
EGDYEDMEILLGSNHDEGTYFLLYDFIDFFEKDGPSFLQREKYHDIIDTIFKNMSRLERDAI
VFQYTNWEHVQDGYLNQKMIGDVVGDYFFICPTNDFAELAAERGMKVYYYYFTHRTST
SLWGEWMGVMHGDEIEYVFGHPLNMSLQFNTRERELSLKVMQTFARFSATGKPVTDDV
NWPLYTRDQPQYFIFNADKNGIGKGPRAQACAFWNDFLPKLRDNAGSAESPCVNTYLSKI
GASSSFTPSCILLLFLIWMLALLGAL
>PverCOE40
MKPRMKNHFRAHQLSVWLRLIPELHRAGMEDVVARHNLFRNHNSADLYDGAVRPDPLS
RVSYYDPTMELVRRRPNSSLTAVEAPTTIESIVTTCISVGGFNHQLFQNQNNATDTLASLEA
AGYAAYSTALSVTIAIGCSLLILNVLIFAGVYYQRDKTRMEVKNLQQQQSRNQAAFETISK
HQHYHMGHSQSSNVIVDVEQDTSAMILAANAHHDIHQPQHLKAPPPSPNTVLQNCLTLPR
KPAMHTYPGHQHGCMTLPKNAGLMNHTACVIAEMQQQQGLVQPPNGNATMASGVPKPP
PPPRARSPESQPLLSSHNNMAQAGLNVPHAAMSEMRV
>PverCOE41
MHFTRNYTKSEMILSETTMIYWSNFARTGNPNEAPEGDASHGGRQERSRFKNIEWTAYEG
VHKKYLNLDTKPKLKNHYRAHRLSFWLNLVPDLHKPGGDDVPASHHELPPDDEPPLPPLP
ALNPRKLPTTESPHQELRTAAGNGTVAVTGNPLDST
>PverCOE42
MLIFAGIYYQRERDRKRQNQARQHHRSNSTPSTEAIPMTQRPSTPGSSARQSPVLTKKDKL
HELPPSYTIVSHSPNCEDRLPATQRSRERPLPPARSSSNPPGNTIKKRVQIQEISV
>PverCOE43
MQMPYTTIADSLTAIFCIMWFFIVVISIEKSEAGVSLLVHKYSTRIVKTKYGPLRGIILHIHPS
VEAFLGIPYATPPVGSLRYMPPVTPSLWRTTRLADRFSPVCPQRLPDISNRTEALLEFPKGH
LLHLEKILPLLANQSEDCLYLNLYVPRTATGGEESSSLPCLVYVHGESYEWSSGNVYDGTL
LASMGRVIVVTINFRLGVLGFVKTGTKGSTQGNFGLMDLMAGLHWLRENLSAFGGDPQR
ITLMGHGTGAALVNFIAVSPVAKELLHRVILLSGSGLSPWALQRDPMWVKRSLAEHTGCH
GDLLEDDLAPCLRLKPLDQLLSVKLEPPRFLPGFAPFIDGTILMNPTTSPPTSSASSIVTTPG
YEMALFPKQDLLFSVTTSEAYLDLTAQDLEFGFNDTKRDRILRTFVRNSYYYHLNEIFSTL
KNEYTDWERPSLNPMNVRDTTLDLLSDGNTVAPLIRIGYLHALNGGRTFFMHFQHHSGIR
DIPMRSGSVKGDDLTFVFGLPLVGDKSPSANNNYSRGDFLVSKTLVNYISNFVRSGDPNQE
TATFSTGGESKNQQSDNQKEPFWDTYDTINQFYMELGSRAERKDHYRGHKMSLWLNLIP
QLHRASDGDDVSMRHHHFQEEDEQYYDGYVRPQLKERPRFVQILASPSEAAKIPTTTTLA
ALPPKSDTALQAITTECPPNNATYVMSSINRNSNNLIRRLANSHFHSYTTALTVTIGVGCFL

LLLNVLIFAGIYHQKSRGKSKEKQKAEIEMGQSENTCSTLLLIFL
>PverCOE44
MYTADAPVWSGVKMADGFGPVCPQSFPDVGKMAPHRKEYFERLQGQLMNQSEDCLYL
NIYAPICDDKSNTRKYPVMVFIHGESYEWNSGNPYDGSVLAAYGNVIVVTINFRLGVLGF
LKAGVESQSKSNFGLVDQVAALLWVKENIAAFKGDANSVTLFGHGTGAVCASLLMISPMI
LKENERLFHRAILMSGTALADWAMAGNPTKVTYQVAKALNCQIHEDFAECLRKKRLDEIL
AAGEVASDYKTRFGPVVDSIVVPNDPKKSMTQYTDLFRRFEVMYGVTELESIDLLGPVAL
VKGLLEKERDQELRAYFHSRCEMKPELCLQRTMDEYAPAHAQSSFTGREPDKASVARDTL
LDILSDARTVAPVLQTGRYHAALNLQSYFYVFTQRTNSKEYIRNKSYNGGELPYVFGVPL
DGPKYHFSDSYTEQEKMFSEIVMMYFTNFAGTGNPNVPKRQIFYSMNTAYWAQFDIEWPE
FDTQEEWYLQLDSPPQPLQHYRDSKIRYWNEIFPKLTENFSYNIDTPTKRTLLVPPRKTPPA
FLYDNLPRRPTVPSYNSYMNFRKPDNIVRNAETVIESPAGEIKEASTMTVVVIVGVVFFLV
NVLLLLTLYFKCYKKKKAAAGSESSGDKKDAGDSVIFSGCSITRLMRKSTRSDDSYAAKT
SQLNRQMSGSTLDAHTKVRQWIAQEIIQKYSPRLCRKTPPSRKNSPGDFALSMNAEGDST
MGRGPTRPASPVEEKAVPMRTSTITRPKVKAPKVSVGVDATPTGRGNKSLDCPSTKSPLRR
SFTLEDFSPRVVNLQEDTRRSATNVSLQQSDFQPTYIKIEHCPSKSDATSTKRLRTFDPNGD
VNVTSREESEAAPQPLSPEESLRVIKRRNCPKVLPDHPAMISKRRSMPVAGCFVPIPESSALP
PKNSSLPKSLNRFPPAPPPRCSTLTKQTSNPLPVSVVLVEQPEEEPEIVCNNLYVGPLIPRKPA
QGGSLGRLERSFPKREGRENEGVLGVGKKSRLPTPAKTAAGDGGGFSKESSSSESSPSEGS
DTGTVVKRMQ
